# Cold Atmospheric Plasma Triggers Apoptosis via the Unfolded Protein Response in Melanoma Cells

**DOI:** 10.3390/cancers15041064

**Published:** 2023-02-07

**Authors:** Tom Zimmermann, Sebastian Staebler, R. Verena Taudte, Sumeyya Ünüvar, Sabine Grösch, Stephanie Arndt, Sigrid Karrer, Martin F. Fromm, Anja-Katrin Bosserhoff

**Affiliations:** 1 Institute of Biochemistry, Friedrich-Alexander-Universität Erlangen-Nürnberg, 91054 Erlangen, Germany; 2 Institute of Experimental and Clinical Pharmacology and Toxicology, University of Erlangen-Nürnberg, 91054 Erlangen, Germany; 3 Core Facility Metabolomics/Mass Spectrometry, Philipps University Marburg, 35043 Marburg, Germany; 4 Institute of Clinical Pharmacology, Faculty of Medicine, Goethe University Frankfurt, 60590 Frankfurt, Germany; 5 Department of Dermatology, University Hospital of Regensburg, 93053 Regensburg, Germany; 6 Comprehensive Cancer Center (CCC) Erlangen-EMN, 91054 Erlangen, Germany

**Keywords:** melanoma, cold atmospheric plasma, apoptosis, unfolded protein response, ceramide

## Abstract

**Simple Summary:**

The development of novel therapies for cancer treatment is the key to improve patient prognosis. Cold atmospheric plasma (CAP) is a promising technology to treat all kinds of cancers, but the exact mechanisms of its action have not yet been identified. This study aimed to understand the effects of CAP treatment on melanoma cells to contribute to its potential use in tumor therapy. We identified a strong activation of the unfolded protein response along with a reduction in intracellular ceramide levels, which were linked to each other and to CAP-induced cell death. Furthermore, we showed that pharmacological reduction in ceramides strengthens the effects of CAP treatment in melanoma cells.

**Abstract:**

Cold atmospheric plasma (CAP) describes a partially ionized gas carrying large amounts of reactive oxygen (ROS) and nitrogen species (RNS). Numerous studies reported strong antitumor activity of CAP, thus rendering it a promising approach for tumor therapy. Although several cellular mechanisms of its cytotoxicity were identified in recent years, the exact molecular effects and contributing signaling pathways are yet to be discovered. We discovered a strong activation of unfolded protein response (UPR) after CAP treatment with increased C/EBP homologous protein (CHOP) expression, which was mainly caused by protein misfolding and calcium loss in the endoplasmic reticulum. In addition, both ceramide level and ceramide metabolism were reduced after CAP treatment, which was then linked to the UPR activation. Pharmacological inhibition of ceramide metabolism resulted in sensitization of melanoma cells for CAP both in vitro and ex vivo. This study identified a novel mechanism of CAP-induced apoptosis in melanoma cells and thereby contributes to its potential application in tumor therapy.

## 1. Introduction

Cold atmospheric plasma (CAP) is a weakly ionized gas with diverse biological and chemical effects. Its main components are reactive oxygen (ROS) and nitrogen species (RNS), as well as other ions and uncharged particles. The exact composition is, however, heterogeneous and may be adapted to the intended use. Early studies on biological CAP effects detected strong antimicrobial activity which allowed efficient decontamination of surfaces and medical instruments [1,2]. In addition, experiments with human cells and tissue did not detect detrimental effects of CAP application [3,4,5], thus enabling the potential treatment of local infections. Several studies and clinical trials have since shown major benefits of CAP in dentistry and dermatology [6,7,8,9,10]. Plasma oncology, referring to CAP treatment of malignant tumors, is a relatively novel field of plasma medicine. It quickly gained interest after several studies found strong antitumor activity of CAP in a variety of tumor entities, while healthy cells remained mostly unaffected [11,12,13,14]. Given the ever-present challenge of therapy resistance in many tumors, the development of novel therapeutic approaches still represents a major target of today’s oncological research [15,16]. Beside the above mentioned antitumor effect and its selectivity for malignant cells, CAP was shown to increase chemosensitivity of resistant tumor cells [17,18]. It therefore represents a promising and quickly evolving field in tumor therapy.

In recent years, cutaneous melanoma has become an important disease of plasma oncology, mainly because the use of CAP for the treatment of this dermal disease has been extensively studied preclinically and safety data are available. Furthermore, the superficial localization of primary tumors allows for non-invasive and repeated treatment in potential clinical applications. Multiple studies have shown the extraordinary responsiveness of melanoma cells to CAP [12,19,20,21]. The exact molecular mechanisms are, however, not fully understood to date. Several mechanisms have been proposed to contribute to CAP-induced apoptosis, mainly involving oxidative stress, DNA damage, and mitochondrial dysfunction [22,23,24]. As these could not entirely explain the strong cytotoxicity of CAP, and most underlying processes and signaling pathways have not been identified with certainty yet, there is a need for further analysis of the molecular effects of CAP treatment. While DNA damage and transcriptional and even epigenetical modification have been investigated extensively [25,26,27], little interest has been given to CAP-dependent changes in protein homeostasis. Recent studies reported a broad range of protein modifications by CAP treatment of biomaterials and food [28,29,30]. Additionally, Krewing et al. found irreversible protein denaturation in prokaryotic *Escherichia coli* to be a major cause of CAP-mediated bacterial inactivation [31]. Eukaryotic cells use the unfolded protein response (UPR) to cope with unphysiological amounts of denatured or misfolded proteins [32]. It prevents further overload of protein folding mechanisms by degrading mRNA, halting translation, and increasing expression of molecular chaperons. If these measures are not sufficient to restore protein homeostasis or have to be sustained for an extended period of time, UPR-mediated apoptosis is induced via transcription factor CHOP [33]. Two studies by Itooka et al. reported UPR activation after CAP treatment of yeast cells, thereby indirectly proving that protein denaturation also occurs in eukaryotic cells [34,35]. Despite being a protective mechanism, increased activity of the UPR was found to contribute to fungicidal effects in these studies. To this day, there is no published evidence that CAP causes protein denaturation and UPR activation in human cells.

Activation of the UPR is thought to be a hallmark of tumor cells and has large impact on malignant transformation, progression, and metastasis [36]. This is mainly due to increased protein folding capacities required for adaption to elevated protein synthesis and metabolic changes. As prolonged UPR signaling results in apoptotic cell death, it is essential for tumor cells to actively suppress the master regulator of UPR-induced apoptosis, CHOP. The exact mechanism of such suppression remains unclear, but reduced stability of CHOP mRNA was detected in transformed fibroblasts [37]. Different approaches have been proposed to exploit the dysregulation of UPR signaling for tumor therapy, mostly based on a normalization of protein folding mechanisms to halt tumor growth [38]. Further stimulation of the UPR to reactivate CHOP might be equally useful to selectively target tumor cells. The aim of this study was to investigate UPR regulation after CAP treatment of melanoma cells and its potential involvement in CAP-mediated apoptosis. 

## 2. Materials and Methods

### 2.1. Chemicals and Solutions

Sources for chemicals used in this study: Amitriptyline (Sigma Aldrich, Steinheim, Germany), BAPTA AM (Merck, Darmstadt, Germany), ceramide C16:0 and ceramide C24:1 (Cayman Chemical, Ann Arbor, MI, USA), DMSO (Sigma Aldrich), dodecane (Thermo Fisher Scientific, Waltham, MA, USA), ethanol (VWR, Radnor, PA, USA), fumonisin B1 (Cayman Chemical), hydrogen peroxide 3% (Sigma Aldrich), PDMP (Enzo Life Sciences, Farmingdale, NY, USA), 4-phenylbutyrate (Cayman Chemical, Ann Arbor, MI, USA), ryanodine (Santa Cruz, Dallas, TX, USA), sodium nitrite (Sigma Aldrich).

### 2.2. Cell Culture

Melanoma cell line Mel Juso (RRID:CVCL_1403) was cultivated in RPMI 1640 Medium with 2% sodium bicarbonate, while the Mel Im cell line (RRID:CVCL_3980) required DMEM low-glucose medium. All cell culture media were supplemented with 1% penicillin/streptomycin and 10% FCS. Cells were incubated at 37 °C and 8% CO_2_. When reaching approximately 80% confluence, they were washed once with PBS and detached using 0.05% trypsin and 0.02% EDTA in PBS. The reaction was stopped using cell culture medium, followed by centrifugation, removal of the supernatant, and resuspension in cell culture medium. Cells were either passaged or counted using a Neubauer counting chamber. Cell lines were provided by the tissue bank of the Institute of Pathology of the University Hospital Regensburg (Regensburg, Germany) and tested for mycoplasma contamination regularly. All cell culture media and chemicals were obtained from Sigma Aldrich.

### 2.3. CAP Treatment

Direct treatment of melanoma cells with SMD-generated CAP was performed using a plasma care^®^ device (terraplasma medical GmbH, Garching, Germany) according to the manufacturer’s instructions, but with a 5 min period of warm up without a sample. A custom, 3D-printed spacer was used to achieve a tight fit of single 35 mm cell culture dishes to the device. Distance between cells and plasma source was approximately 10 mm, which is comparable to previous studies using similar technology [39]. For treatment, approximately 200,000 cells were seeded in 35 mm cell culture dishes and incubated overnight. Cells were washed once with PBS, which was then removed completely to allow direct application of CAP to the cells. All samples were treated for 2 min, followed by addition of cell culture medium and incubation for 1 h at 37 °C and 8% CO_2_. Control cells were prepared in a similar way, but instead of CAP treatment, they were incubated for 2 min without medium to ensure comparability.

### 2.4. Application of Inhibitors

Unless otherwise stated, treatment with inhibitors began 24 h after seeding 200,000 cells in 35 mm cell culture dishes. The treatment procedure included 1 h of preincubation with each inhibitor, followed by CAP treatment and application of the inhibitor for one more hour. Preincubation was reduced to 30 min when using inhibitors of calcium metabolism (Ryanodine, BAPTA AM) to ensure comparability with previous studies [40]. PDMP was applied for a total of 48 h, starting with the seeding process and including a full change of cell culture medium and inhibitor after 24 h. Due to this increased incubation time, only 100,000 cells were seeded in 35 mm cell culture dishes for this experiment. In control experiments, cells were treated equally and received an appropriate amount of solvent.

### 2.5. Treatment with Nitrite and Hydrogen Peroxide

Application of acidified nitrite was previously described [39]. Similar to this study, extracellular solution (ECS) containing 145 mM NaCl, 5 mM KCl, 10 mM glucose, 1.25 mM CaCl_2_, 1 mM MgCl_2_, and 10 mM HEPES was used to dilute sodium nitrite and hydrogen peroxide. Approximately 200,000 cells/well were seeded to 6-well plates one day before treatment. Cells were washed with PBS before each solution was applied for exactly 5 min. After removal of nitrite and hydrogen peroxide solutions, cells were incubated for 1 h in cell culture medium at 37 °C and 8% CO_2_.

### 2.6. Western Blot Protein Analysis

Cells were detached, washed, and immediately lysed using radio-immunoprecipitation assay buffer (Roche, Basel, Switzerland). Protein concentration was determined by Pierce™ BCA protein assay (Thermo Fisher Scientific). Approximately 20 µg of protein were loaded on 10% SDS polyacrylamide gels for electrophoresis and subsequently blotted on PVDF membranes (Bio-Rad, Hercules, CA, USA). Ponceau S staining was used to validate equal protein load. Membranes were then blocked using 5% non-fat dried milk (NFDM) in TBST for 1 h. Primary antibodies against phospho-eIF2α (1:500 in 5% NFDM, ab32157, Abcam, Cambridge, UK), eIF2α (1:1000 in 5% NFDM, ab5369, Abcam), and ATF6 (1:1000 in 5% NFDM, sc-166659, Santa Cruz) were applied overnight at 4 °C. Following several washing steps with TBST, membranes were incubated with horseradish peroxidase (HRP)-coupled secondary antibodies (1:2000 in TBST, 7074 and 7076, Cell Signaling, Danvers, MA, USA) for 1 h at room temperature. Detection of proteins was realized by addition of Clarity™ Western ECL Substrate (Bio-Rad) and subsequent visualization using a Chemostar chemiluminescence imager (Intas, Goettingen, Germany). Quantitative analysis of signal intensity was performed with LabImage software (version 4.2.3, Kapelan Bio-Imaging GbmH, Leipzig, Germany). Original blots see File S1.

### 2.7. Analysis of mRNA Expression Using Real-Time PCR

Total RNA was isolated using E.Z.N.A.^®^ Total RNA Kit II (Omega Bio-Tek, Norcross, GA, USA) in accordance with the manufacturer’s instructions, followed by cDNA generation as described elsewhere [41]. Real-time PCR was then performed using LightCycler^®^ 480 II devices (Roche). Forward and reverse primers were obtained from Sigma Aldrich, and their sequences are shown in Table 1.

### 2.8. Overexpression and Analysis of GFP

Approximately 100,000 cells were seeded to 35 mm cell culture dishes and incubated overnight. On the next day, they were transfected with 0.5 µg of plasmid DNA (pCDH-CMV-MCS-EF1-copGFP) using lipofectamine LTX (Thermo Fisher Scientific) according to the manufacturer’s instructions. After 24 h, cells were treated with CAP as described above and immediately monitored using an Olympus IX83 inverted microscope in combination with Olympus CellSens Dimension software (version 2.3, Olympus, Tokyo, Japan). Exposure times had to be adjusted to compensate for reduced signal intensity after CAP treatment, accompanied by necessary adjustments of brightness and contrast. As a consequence, signal intensity is not comparable between and within treatment groups and cannot be taken into consideration. Only the aggregation of GFP signal, as characterized by spots of larger intensity as compared to surrounding areas, was assessed here and evaluated manually. At least 30 cells per treatment group and biological replicate were analyzed.

### 2.9. siRNA-Mediated Knockdown

Approximately 200,000 cells were seeded to 35 mm cell culture dishes and immediately transfected with 10 pmol siRNA pool against CHOP (siTools Biotech GmbH, Planegg, Germany) using lipofectamine RNAiMAX (Thermo Fisher Scientific). Following an incubation period of 24 h, cells were treated with CAP as described above.

### 2.10. Quantification of Apoptotic Cells

Apoptosis was detected by Annexin V-FITC and propidium iodide (PI) staining using flow cytometry. After treatment and incubation, cells were detached as described above. Both cell culture medium and PBS used for washing were collected and combined with the cell suspension. Cells were then washed once with PBS and immediately stained using Annexin V-FITC Apoptosis Kit (MyBioSource, San Diego, CA, USA) according to manufacturer’s instructions. Samples were analyzed using a BD LSRFortessa™ flow cytometer in combination with BD FACSDiva™ software (Version 8.0, BD Biosciences, San Jose, CA, USA).

### 2.11. Liquid Chromatography-Mass Spectrometry Analysis

Melanoma cells underwent CAP treatment for 2 min and subsequent incubation for 1 h. The medium was removed and the cells washed twice with PBS. Cells were detached using a cell scraper and transferred to Eppendorf tubes. In one aliquot of each sample, cell count was determined using a Neubauer chamber, which was used for data normalization. The remaining cells were lysed using ice-cold 80% methanol. Cell lysates were centrifuged and the supernatant transferred to HPLC vials. Metabolite extracts were dried under a gentle stream of nitrogen at 30 °C and stored at −80 °C until analysis. Prior to analysis, samples were reconstituted in eluent. Samples were analyzed using an Ultimate 3000 liquid chromatograph (LC) hyphenated to a QExactive Focus mass spectrometer (MS) with a heated electrospray ionization source (DFG-funded INST 90/1048-1 FUGG). Each sample was analyzed in four LC-MS conditions: hydrophilic interaction liquid chromatography (HILIC) with positive and negative polarization and reverse phase chromatography with positive and negative polarization. Analyte separation in HILIC was achieved on an Acquity UPLC BEH Amide, 1.7 µm, 2.1 × 100 mm column, whereas RP separation was achieved on a Acquity UPLC BEH C18, 1.7 µm, 2.1 × 100 mm column with guard columns installed for both conditions (all columns from Waters, Eschborn, Germany). Column temperature was 40°C, the flow rate 0.35 mL/min, and the injection volume 2 µL for all analyses. The HILIC eluent was A = 10 mM ammonium formate (LC-MS grade; Merck, Darmstadt, Germany), pH = 3.5 (formic acid), B = 5% H_2_O, 95% ACN (all three LC-MS grade; VWR chemicals, Darmstadt, Germany), 10 mM ammonium formate. The gradient program was 100% B (0–2 min) decreased to 30% B (14 min), isocratic conditions with 30% B (16.5 min), increased to 100% B (17.5 min). The RP eluent was A = water, 0.1% FA and B = methanol, 0.1% FA. Chromatographic separation was achieved using a gradient program: 10% B (0 min) increased to 98% B (9 min), isocratic conditions at 98% B (11 min), back to 10% B (15 min). Mass spectrometric conditions included a capillary voltage of −3 kV in negative mode and 3 kV in positive mode, capillary temperature of 380 °C, and the auxiliary gas temperature was 400 °C. The sheath gas pressure, auxiliary gas pressure, and sweep gas flow rate were set to 60, 20, and 0 arbitrary units, respectively. Nitrogen 5.0 was used for these gases. The scanning range was 66.7 to 1000 *m*/*z* in both ionization modes. Furthermore, the mass spectrometer was operated in discovery acquisition mode with the instrument switching between full scan and ddMS2 mode. Resolution of the analyzer in full scan was set to 70,000 and the maximum inject time set at auto with an AGC target of 1,000,000 1e6. In ddMS2, the resolution was set to 35,000 with the maximum injection time set to auto and an AGC target of 50,000. For data analysis, TraceFinder 4.1 and Compound Discoverer 3.1 were used. 

### 2.12. Addition of Ceramides

Ceramides were dissolved in a mixture of 98% ethanol and 2% dodecane, which was reported to increase the solubility and cellular uptake of ceramides [42,43]. Cells were incubated for 2 h with cell culture medium containing 0.2% BSA instead of 10% FCS, followed by the addition of ceramides with a final concentration of 20 µM. After incubation for 1 h, CAP treatment was performed as described above followed by another hour of incubation with ceramides in serum-free medium. Intracellular delivery of ceramides was not validated directly. However, as ceramide-containing medium was completely removed during CAP treatment, no ceramides were present in the extracellular space. This was done to prevent false-positive results due to scavenging or quenching of reactive species outside of the cell. 

### 2.13. Animals, Preparation, and Treatment of Murine Tissue

The Tg(Grm1)EPv transgenic mice were originally established by Suzie Chen (Department of Chemical Biology, Rutgers University, Piscataway, NJ, USA) and Jürgen Becker (Medical University of Graz, Department of General Dermatology, Graz, Austria). Mice were bred on a C57BL/6J background and housed in groups of up to 5 animals per cage, had ad libitum access to water and food, and were kept under standardized conditions at a temperature of 20–22 °C, relative humidity of 47–48%, and a 12 h light–dark cycle. Animal care and the experimental procedures were carried out in accordance with guidelines of the German law governing animal care. Breeding of tumor-bearing mice was approved by the Ethics Committee for Animal Research of the Bavarian government. We used mice at an age of approximately 130 to 150 days to obtain primary tumors large enough for tumor resection. On the day of the experiment, mice were sacrificed by cervical dislocation before the whole tumor-bearing tail was removed. Superficial skin and parts of the tumor mass were removed surgically. Tail regions that had tumors were then dissected and roughly cut to a length of 6–8 mm. Treatment was similar to in vitro experiments; a short washing step with prewarmed PBS was followed by the addition of 100 µM amitriptyline in DMEM high-glucose cell culture medium for 1 h at 37 °C and 8% CO_2_. Afterwards, tail sections were washed again and placed in a 35 mm cell culture dish with the resection wound facing the CAP device. To ensure comparability of results, all samples of a biological replicate were obtained from the same animal and treated simultaneously in the same cell culture dish. Following CAP treatment for 2 min, tail sections were placed in fresh DMEM high-glucose medium with 100 µM amitriptyline for another hour at 37 °C and 8% CO_2_. In the next step, tail sections were washed with PBS and underwent fixation using 4% PFA/PBS for 24 h at 4 °C. They were then washed for 10 min with PBS and decalcified using 14% EDTA on a shaker at 4 °C for a total of 12 days. During this time, EDTA solution was replaced in 48 h intervals. After decalcification was performed, tail sections were dehydrated and embedded in paraffine (Paraplast plus, Sigma Aldrich, Germany). Histological sections of 5 µm thickness were cut using a sliding microtome (Microm GmbH, Walldorf, Germany).

### 2.14. Immunofluorescent Stainings

Prior to immunofluorescent stainings, histological tissue sections were rehydrated and bleached to remove the strong pigmentation of tumor cells. A bleaching solution of 1% dipotassium phosphate, 0.5% potassium hydroxide, and 3% hydrogen peroxide was applied for 1 h at room temperature and eventually stopped using 1% acetic acid. The slides were then washed with double-distilled water before staining began. First, antigen retrieval was performed using 20 µg/mL proteinase K for 10 min at room temperature. This was followed by permeabilization with 0.3% Triton-X100/PBS for 5 min and blocking by application of 1% BSA/PBS for 1 h. The tissue section were incubated with the primary antibody against cleaved caspase 3 (1:400 in 1% BSA/PBS, 9664, Cell Signaling) overnight at 4 °C. On the next day, slides were incubated with the secondary antibody coupled to AlexaFluor™ 555 (1:400 in 1% BSA/PBS, A32732, Thermo Fisher Scientific) for 1 h at 37 °C. In the final step, nuclei were stained with DAPI (1:10,000 in PBS, Sigma Aldrich) and eventually mounted using Aqua-Poly/Mount (Polysciences Inc., Warrington, PA, USA). Analysis of immunofluorescent staining was realized with an Olympus IX83 inverted microscope and Olympus CellSens Dimension software. Total melanoma cells were counted using DAPI staining. Cells were considered positive for cCasp3 if nuclear staining was of higher intensity than cytoplasmic staining. This was counted manually within a 200 µm margin starting from the surface of the resection wound. For each treatment group and biological replicate, 5 images with a total of approximately 1500 cells were analyzed. H&E stainings were performed following standard protocols.

### 2.15. Statistical Analysis

All experiments included at least three biological replicates. Statistical analysis was performed using GraphPad Prism 9 (Version 9.1.2., GraphPad Software Inc., San Diego, CA, USA). Experiments with two groups were analyzed using two-tailed Student’s *t*-test, while more than two groups were compared by one-way analysis of variance (ANOVA) followed by Tukey’s HSD post hoc tests. Statistical analysis of Figure 2A, which includes multiple variables, was performed using two-way ANOVA followed by Sidak’s multiple comparison tests. All bar graphs are shown as mean ± standard error of the mean (SEM). A critical value of *p* < 0.05 was set for statistical significance and is denoted within figure legends. We assumed data distribution to be normal but did not formally test for it.

## 3. Results

### 3.1. Cold Atmospheric Plasma Causes Activation of the Unfolded Protein Response in Melanoma Cells

**Figure 1:**
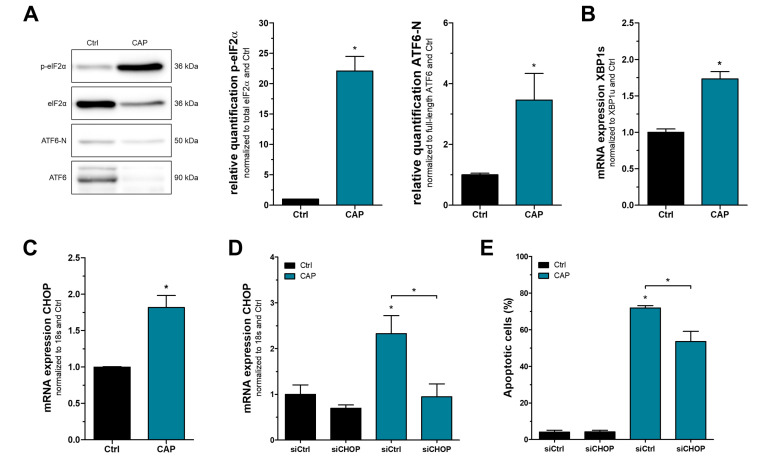
CAP treatment of melanoma cells activates the UPR. (**A**) Western blot analysis of phosphorylated eIF2α in comparison to total eIF2α protein, as well as activated ATF6-N and its inactive variant ATF6 in Mel Juso cells. (**B**,**C**) Expression analysis of spliced XBP1s with unspliced XBP1u as reference and mRNA expression of pro-apoptotic CHOP. (**D**) Expression of CHOP mRNA and (**E**) quantification of apoptotic cells in melanoma cells after siRNA-mediated knockdown of CHOP for 24 h and subsequent CAP treatment. Only relevant statistical comparisons are shown in the graphs, including Ctrl siCtrl vs. CAP siCtrl, as indicated by asterisks directly above error bars, and CAP siCtrl vs. CAP siCHOP, as shown by the bracket (*n* = 4 in Figure 1C, all other experiments *n* = 3, * *p* < 0.05). All original Western blot images can be found in the Supplementary Materials.

Previous studies described severe cytotoxicity of surface micro-discharge (SMD)-generated CAP on melanoma cells [12]. Here we used a treatment duration of two minutes, which was sufficient to induce apoptosis in melanoma cells. To identify early mechanisms and cellular signaling involved in CAP cytotoxicity, all data of this study were generated exactly one hour after CAP treatment. First, we assessed the consequences of CAP on the unfolded protein response (UPR) by analyzing its three main signaling pathways PERK (PKR-like endoplasmic reticulum kinase), ATF6 (Activating transcription factor 6), and IRE1 (Inositol-requiring enzyme 1) in the melanoma cell line Mel Juso. Activity of the protein kinase PERK was indirectly quantified using phosphorylated eIF2α (Eukaryotic translation initiation factor 2 subunit alpha) and showed a strong increase after the CAP treatment of the melanoma cell line Mel Juso (Figure 1A). ATF6 is cleaved proteolytically to yield the active form ATF6-N. While we could not detect elevated levels of ATF6-N, there was a strong decrease in full-length ATF6, resulting in a higher active-to-inactive ratio as compared to control cells (Figure 1A). The third signaling pathway of the UPR starts with activation of IRE1 and leads to splicing of XBP1. We analyzed the ratio of spliced XBP1s and unspliced XBP1u and found a significant increase in XBP1 splicing after CAP treatment (Figure 1B). Similar effects were observed in the melanoma cell line Mel Im, but without the aforementioned activation of ATF6 (Figure S1A,B). We thereby conclude that CAP treatment causes an activation of UPR signaling pathways PERK and IRE1, while a regulation of ATF6 was not reliably detected. In the next step, we analyzed mRNA expression of the pro-apoptotic transcription factor CHOP, which is thought to be the major consequence of strong UPR activation. A significant increase was found in both cell lines, indicating that CAP-induced apoptosis might be partially mediated by UPR activation (Figure 1C and Figure S1C). Finally, we performed an siRNA-mediated knockdown of CHOP to validate its contribution to CAP-induced apoptosis (Figure 1D,E and Figure S1D,E).

**Figure 2:**
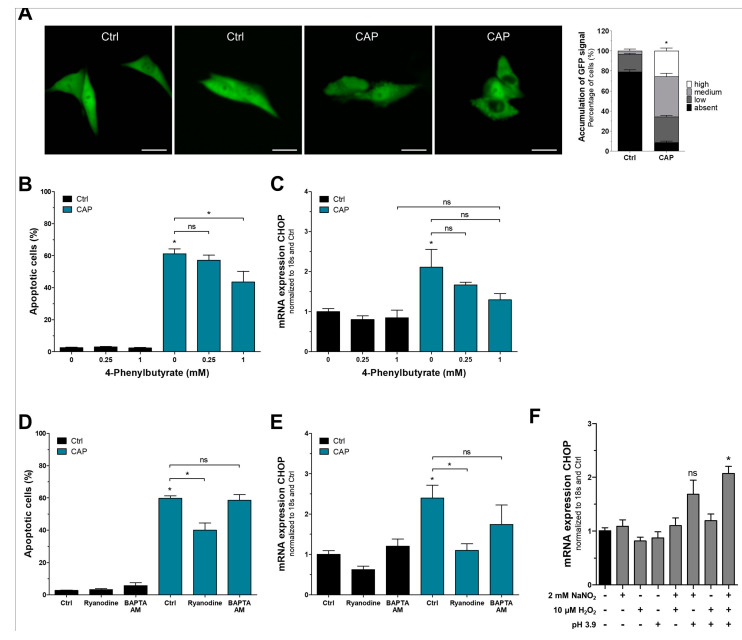
Protein aggregation and calcium release from the endoplasmic reticulum are responsible for CAP-mediated activation of the UPR. (**A**) Representative images of GFP accumulation in untreated (Ctrl) and CAP-treated cells directly after treatment. Brightness and contrast are not comparable as exposure times had to be adjusted to compensate for CAP-induced photobleaching. Scale bars are 20 µm. GFP accumulation was scored manually and is shown on the right side. (**B**,**C**) Flow cytometric analysis of apoptotic cells, as well as mRNA analysis of CHOP expression after treatment with chemical chaperon 4-phenylbutyrate. (**D**,**E**) Similar analyses after treatment with ryanodine, an inhibitor of intracellular RyR calcium channels, and calcium chelator BAPTA AM. (**F**) Expression analysis of CHOP after treatment of Mel Juso cells with different combinations of nitrite, hydrogen peroxide, and extracellular acidification (*n* = 3, * *p* < 0.05, ns = not significant).

We then aimed at investigating the reason for UPR activation in response to CAP. Mel Juso cells were transiently transfected with a plasmid expressing GFP under the control of an EF1 promoter, resulting in a homogenous cytoplasmic signal. We detected increased GFP accumulation directly after CAP treatment, possibly due to changes in protein structure and folding (Figure 2A), which might be responsible for UPR activation. In order to validate this hypothesis, the chemical chaperon 4-phenylbutyrate was used to stabilize protein folding and potentially mitigate CAP-induced cytotoxicity. When assessing apoptosis via flow cytometry, we found the percentage of apoptotic cells to be significantly reduced after combining CAP with 4-phenylbutyrate compared to CAP alone, which shows that CAP-induced protein misfolding contributes to its cytotoxicity (Figure 2B). This is further supported by slightly reduced expression of CHOP after treatment with 4-phenylbutyrate (Figure 2C). A main feature of the UPR is increased expression and activity of molecular chaperons to clear misfolded proteins and restore protein homeostasis. Given that the majority of these chaperons are dependent on calcium [44], which is released from the endoplasmic reticulum during CAP treatment [40], we were interested whether this calcium loss impairs chaperon function and thereby prevents recovery of misfolded proteins. We used ryanodine to inhibit ryanodine receptors and thereby calcium release from the endoplasmic reticulum. The resulting calcium retention significantly attenuated CAP-induced apoptosis (Figure 2D) and CHOP expression (Figure 2E). In addition, calcium chelator BAPTA AM was used to remove free calcium ions from the cytoplasm, which did not alter the percentage of apoptotic cells or CHOP expression after CAP treatment (Figure 2D,E). Our group recently published data highlighting the cytotoxic properties of CAP-derived RNS [39]. For identification of the reactive species responsible for CAP-mediated UPR activation, melanoma cells were treated with nitrite, hydrogen peroxide, and acidic pH either alone or in combination. The exact concentrations and reaction conditions were derived from a two-minute CAP treatment as used throughout this study. Synergistic effects of nitrite and acidic pH could be observed, while hydrogen peroxide contributed only slightly (Figure 2F). 

### 3.2. Ceramides and Ceramide Metabolism Are Affected by CAP Treatment

**Figure 3:**
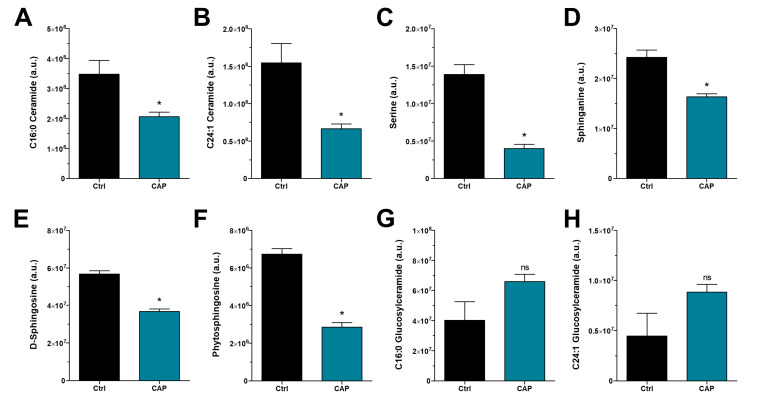
Reduction in ceramides and ceramide metabolism after CAP treatment of melanoma cells. Semiquantitative measurement of metabolites using LC-MS analysis of CAP-treated Mel Juso cells. (**A**,**B**) Two ceramides were assessed, along with substances of (**C**,**D**) de novo ceramide synthesis and (**E**,**F**) salvage pathway. (**G**,**H**) Relative quantification of glucosylceramides (*n* = 3, * *p* < 0.05, ns = not significant). Note: Ceramide annotations are putative.

Activation of the UPR was previously linked to alterations of sphingolipid levels, with both an increase [45,46] and reduction [47,48] in intracellular ceramides causing increased UPR signaling. Consequently, we assessed intracellular levels of ceramides and their metabolites using coupled liquid chromatography and mass spectrometry (LC-MS). The main cellular ceramides C16:0 and C24:1 were significantly downregulated after CAP treatment (Figure 3A,B), indicating that the cells underwent major changes in ceramide homeostasis. We then analyzed serine and sphinganine, two molecules that are involved in de novo ceramide synthesis, which were downregulated as well (Figure 3C,D). In order to evaluate ceramide degradation, levels of sphingosine and phytosphingosine were measured. Both molecules are products of ceramidase activity and might enter the salvage pathway to regenerate ceramide. CAP treatment caused a similar reduction in both molecules (Figure 3E,F). Finally, we detected a small and non-significant tendency towards upregulated glucosylceramides (Figure 3G,H), suggesting that ceramide glycosylation might be affected by CAP as well.

### 3.3. Increased Ceramide Levels Attenuate CAP-Induced Apoptosis and UPR Activation

**Figure 4:**
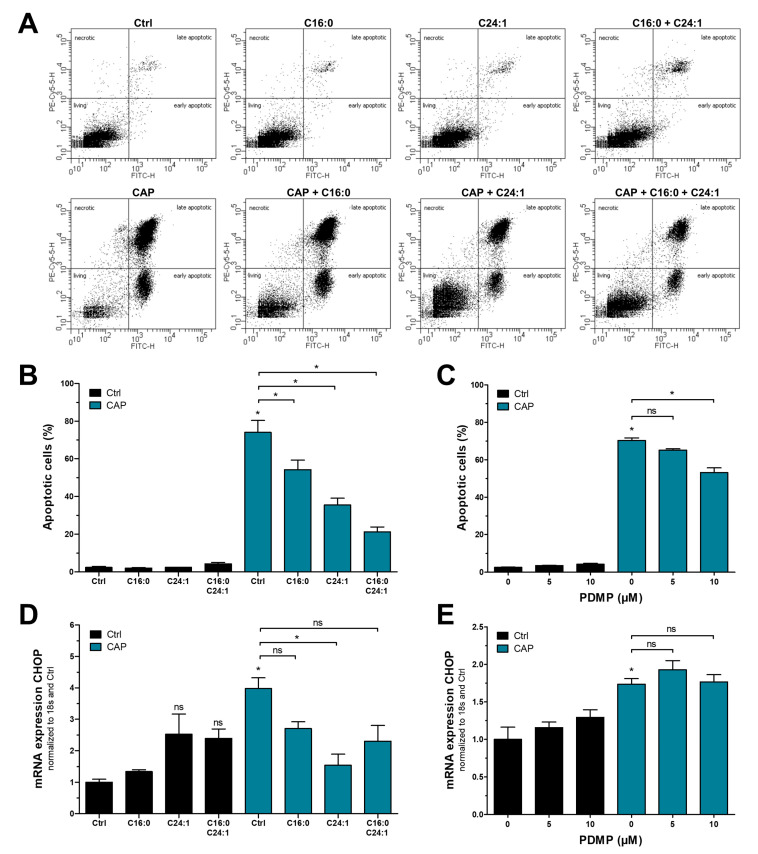
Elevated ceramide levels attenuate CAP-mediated apoptosis and UPR activation. (**A**) Representative examples of flow cytometric detection of apoptotic cells after addition of C16:0 and C24:1 ceramides and treatment with CAP and (**B**) quantification of these cells. (**C**) Quantification of apoptotic cells after combined treatment with PDMP, an inhibitor of glucosylceramide synthase and CAP. (**D**,**E**) Expression analysis of CHOP mRNA in the same settings (*n* = 3, * *p* < 0.05, ns = not significant).

Since CAP treatment of melanoma cells resulted in the downregulation of C16:0 and C24:1 ceramides, we next aimed to modulate CAP effects by increasing ceramide levels. Both ceramides were applied to the cells for one hour before and after CAP treatment at a final concentration of 20 µM. During CAP treatment, however, ceramide solutions were completely removed to prevent scavenging or quenching of reactive species outside of the cell. This treatment resulted in a significant reduction in apoptosis, which was even stronger when both ceramides were combined (Figure 4A,B). Both melanoma cell lines differed in their sensitivity to ceramides. While their addition did not cause apoptosis in Mel Juso control cells (Figure 4B), the same treatment resulted in a slight but non-significant increase in apoptotic cells in Mel Im controls (Figure S2A,B). The aforementioned reduction in CAP-induced apoptosis was, however, detectable in both cell lines. Next, we were interested in a potential link between ceramide reduction and UPR activation. We therefore assessed CHOP mRNA expression after the addition of ceramides and found a similar mitigation of CAP effects, thereby indicating that the CAP-induced activation of UPR signaling is mediated by a loss of ceramides (Figure 4D and Figure S2D). This is even more surprising if the sole effect of ceramide addition without CAP is taken into account: the combined application of C16:0 and C24:1 ceramides caused a slight increase in CHOP mRNA expression in Mel Juso cells and a strong increase in Mel Im cells, which was completely reversed after CAP treatment. The ceramide analog PDMP was used for validation of these findings, as it increases endogenous ceramide levels by inhibition of glucosylceramide synthase. We found a dose-dependent decrease in CAP-induced cytotoxicity, which was present in both cell lines (Figure 4C and Figure S2C). This was, however, not accompanied by reduced CHOP mRNA expression, indicating that the biological function of ceramides during CAP treatment is not limited to the UPR (Figure 4E and Figure S2E). 

### 3.4. Pharmacological Inhibition of Ceramide Metabolism Sensitizes Melanoma Cells for CAP Treatment In Vitro and Ex Vivo

**Figure 5:**
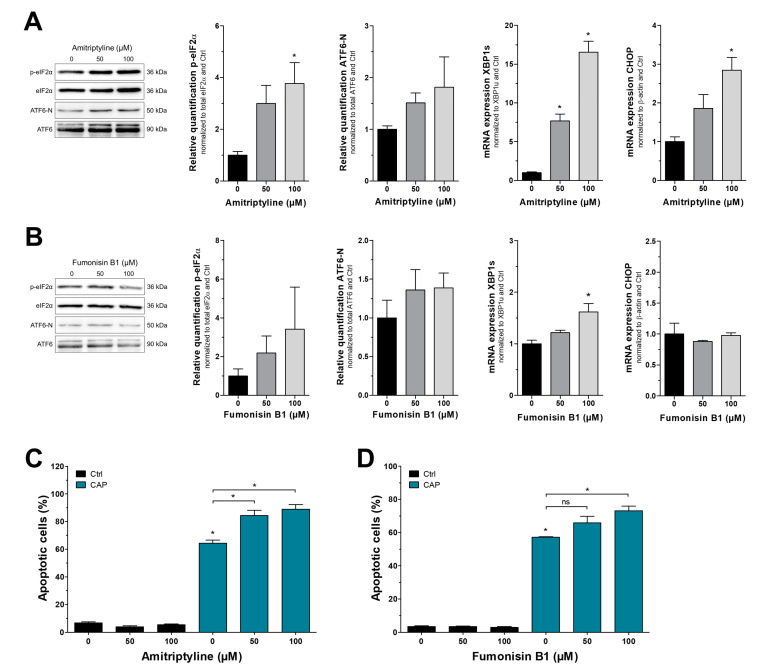
Pharmacological inhibition of ceramide metabolism sensitizes for CAP treatment. Treatment of Mel Juso cells with (**A**) amitriptyline, an inhibitor of acid sphingomyelinase, and (**B**) fumonisin B1, which inhibits ceramide synthases. UPR activation was assessed using several protein and mRNA markers. (**C**,**D**) Flow cytometric analysis of apoptosis using the same inhibitors in combination with CAP treatment (*n* = 3, * *p* < 0.05, ns = not significant). All original Western blot images can be found in the Supplementary Materials.

Since CAP treatment of melanoma cells caused a loss of ceramides and UPR activation at the same time, we next addressed whether a loss of ceramides is sufficient to induce UPR signaling. Amitriptyline is an inhibitor of the acid sphingomyelinase and thereby prevents ceramide generation from sphingomyelin [49]. Melanoma cells were treated for one hour with different concentrations of amitriptyline, leading to activation of PERK and IRE1 signaling as measured by phosphorylated eIF2α and spliced XBP1, respectively (Figure 5A and Figure S3A). Similar to CAP treatment, increased ATF6 signaling was not reliably detected. However, UPR activation resulted in elevated CHOP mRNA expression after amitriptyline treatment (Figure 5A and Figure S3A). A second inhibitor of ceramide metabolism, fumonisin B1, targets ceramide synthases to block both de novo synthesis and the salvage pathway [50]. We applied it similarly to amitriptyline but found fewer consequences of the treatment, as only XBP1s was increased. Phosphorylated eIF2α, ATF6-N, and CHOP were not altered by fumonisin B1 (Figure 5B and Figure S3B) within the assessed period of time, indicating that CAP-mediated UPR activation is not solely due to ceramide reduction. However, since pharmacological inhibition of ceramide metabolism caused activation of the UPR to some degree, we assumed that it might boost CAP-induced cytotoxicity. In order to address this hypothesis, melanoma cells were treated with each inhibitor for one hour before and after CAP. Amitriptyline alone did not trigger apoptosis in Mel Juso cells, despite the aforementioned increase in CHOP expression, but caused limited cytotoxicity in Mel Im. When combined with CAP treatment, amitriptyline led to a dose-dependent increase in apoptotic cells, rendering almost 100% of cells apoptotic (Figure 5C and Figure S3C). Although fumonisin B1 had fewer effects when applied alone, it caused a similar sensitization for CAP in both cell lines (Figure 5D and Figure S3D). This indicates a novel mechanism of the CAP-induced reduction in ceramide levels resulting in UPR-induced apoptosis.

**Figure 6:**
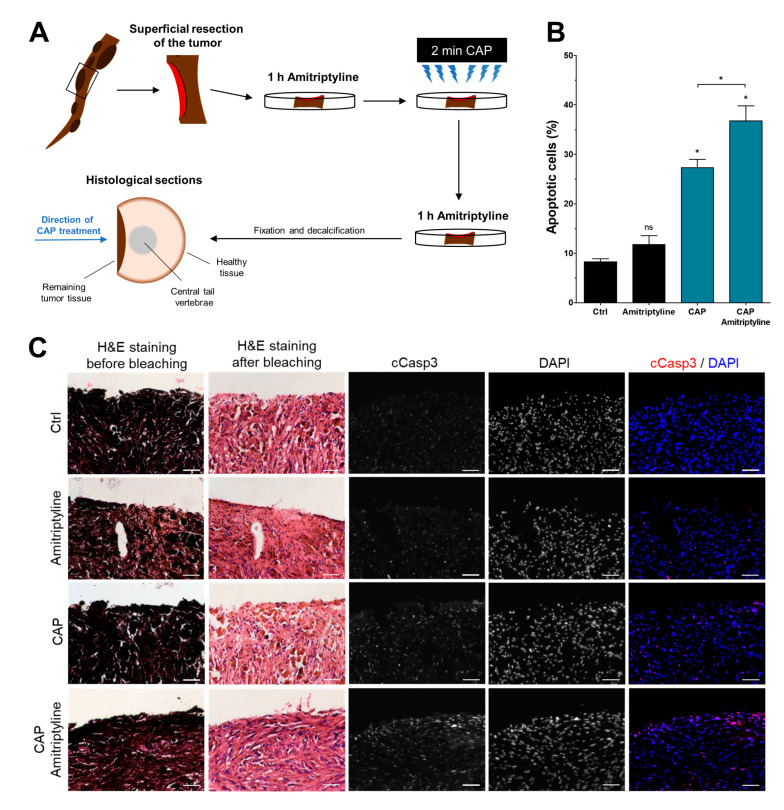
Amitriptyline sensitized melanoma cells for CAP treatment ex vivo. (**A**) Schematic illustration of the experimental setup. Primary melanomas from a Tg(Grm1)EPv mouse model were used for this ex vivo analysis. An illustrated histological section is shown to display the direction of CAP treatment. Bleaching of tissue sections was required to remove strong pigmentation of tumor cells, which would potentially interfere with further analysis. (**B**) Quantification of cells positively stained for cleaved caspase 3 (cCasp3). (**C**) Representative images of hematoxylin and eosin (H&E) staining before and after bleaching, as well as immunofluorescent staining of cCasp3 in combination with nuclear staining using DAPI. Scale bars equal 50 µm (*n* = 3, * *p* < 0.05, ns = not significant).

Since the sensitization of melanoma cells for CAP treatment represents a promising therapeutic approach, we aimed at validating these findings ex vivo. Due to the stronger effects of amitriptyline as compared to fumonisin B1 in vitro, we conducted the following experiment with amitriptyline. We used tumor tissue derived from the tails of Tg(Grm1)EPv mice, a melanoma mouse model derived from C57BL/6 mice that carries a metabotropic glutamate receptor 1 (Grm1) transgene under the control of the dopachrome tautomerase promoter, resulting in melanocyte-specific overexpression [51]. Transgenic mice show melanocytic hyperproliferation and develop primary melanoma in hairless skin areas with 100% penetrance. After sacrificing the mice, we used the tumor-bearing tails and surgically removed superficial skin and primary tumor tissue to resemble tumor resection. Each tissue sample was immediately treated with amitriptyline, CAP, or a combination of both. Treatment conditions were identical to in vitro studies, but only the highest amitriptyline concentration of 100 µM was used. Experimental procedures are illustrated in Figure 6A. For histological analysis, tissue sections were bleached and stained for cleaved caspase 3 (cCasp3) to identify apoptotic cells. Positively stained nuclei were quantified manually, showing a significant induction of apoptosis after CAP treatment (Figure 6B,C). Amitriptyline treatment had no effects on its own, but its combination with CAP treatment increased the number of cells with nucleic cCasp3 even further. Pharmacological inhibition of ceramide metabolism was therefore able to enhance cytotoxic CAP effects not only in vitro, but also ex vivo.

## 4. Discussion

Activation of the UPR is thought to be a major contributor to malignant transformation, tumor progression, and metastasis. Consequently, interfering with UPR signaling represents a promising approach for oncologic therapy, as both normalization and further stimulation of this pathway might lead to antitumoral effects. Recent publications on food processing reported alterations of protein folding as a consequence of CAP treatment [30], which led us to the hypothesis that similar processes potentially activate the UPR in tumor cells and contribute to CAP-induced apoptosis. When assessing established markers of UPR signaling, we found a drastic increase in PERK and IRE1 activity along with increased CHOP expression. Several studies focused on CAP-induced protein modifications but could not provide final conclusions. For example, carbonyl groups were found to be elevated after CAP treatment [28,52], while sulfhydryl groups were increased in one study [53], but reduced in others [52,54]. Such structural changes probably result in conformational changes, exposure of hydrophobic regions, and aggregation of proteins. Cellular mechanisms that counteract proteotoxic stress, such as the UPR, rely on chaperons to restore physiological protein folding. These molecules, however, are strongly dependent on calcium ions and lose their substrate binding ability when deprived of this cofactor [44]. We previously reported the release of calcium from the endoplasmic reticulum to be a major contributor to CAP-induced cytotoxicity [40]. This is validated by our finding that retaining calcium in the endoplasmic reticulum attenuated apoptosis and CHOP expression after CAP treatment. Based on these findings, we hypothesize that CAP results in protein aggregation and thereby activates the UPR. At the same time, protein folding mechanisms are impaired and cannot resolve the proteotoxic stress, leading to even stronger UPR activation. Apoptosis is eventually induced via the UPR-specific transcription factor CHOP. Our findings that UPR activation was mainly due to a synergistic effect of nitrite and extracellular acidification match a previous study that identified this combination to be a major contributor to CAP-induced cytotoxicity in melanoma cells [39]. We therefore propose that the cytotoxic properties of acidified nitrite solutions, which were also described in another study [55], are mediated by UPR activation to some degree.

When screening for underlying mechanisms of CAP-induced activation of the UPR, we detected profound downregulation of ceramide metabolism, including a significant reduction in C16:0 and C24:1 ceramide. This was quite unexpected, as ceramides are widely considered to be pro-apoptotic molecules [56,57] and positively regulate the UPR [45,46]. However, Senkal et al. reported anti-apoptotic effects of C16:0 ceramide in human head and neck squamous cell carcinoma (HNSCC) cell lines [47]. In their studies, loss of C16:0 ceramide resulted in calcium release from the endoplasmic reticulum and subsequent UPR activation via ATF6 followed by CHOP-induced apoptosis [47,48]. The cellular response to CAP treatment, as analyzed here, was remarkably similar, although UPR activation mainly involved PERK and IRE1 instead of ATF6. Many ceramides, including the C16:0 and C24:0 variants, are elevated in melanoma cells when compared to normal cells [58]. Even though levels of C24:1 ceramide were not assessed in the cited study, it highlights that ceramides might in fact have anti-apoptotic properties. When we added ceramides directly to melanoma cell cultures, some degree of cytotoxicity and CHOP activation was detectable. The opposite effect was found when the same ceramides were combined with CAP treatment, now resulting in reduced apoptosis and CHOP expression. It therefore seems likely that C16:0 and C24:1 ceramides may be both pro- and anti-apoptotic, depending on the cellular and experimental context. We also assessed central molecules of de novo ceramide synthesis and salvage pathway, which were significantly reduced after CAP treatment. However, CAP-mediated modulations of ceramide metabolism are probably not limited to reduced synthesis but might also include oxidative modification or degradation of ceramides, as described in normal cells [59,60]. The reported reduction in precursor molecules might also be due to increased ceramide metabolism as an effort to compensate for CAP-mediated ceramide loss. However, this remains hypothetical, and the exact mechanisms of ceramide reduction are yet to be identified.

We then used pharmacological inhibitors of ceramide metabolism to lower endogenous ceramide levels in an attempt to reproduce CAP-mediated UPR activation. Amitriptyline, an inhibitor of acid sphingomyelinase, had quite similar effects, while fumonisin B1 caused less UPR signaling when applied on melanoma cells. A possible explanation can be found in the affected metabolic pathways; while the acid sphingomyelinase directly catalyzes the generation of ceramide from sphingomyelin in a one-step process, fumonisin B1 inhibits ceramide synthases. These enzymes are part of multi-step metabolic pathways that generate ceramide from serine or sphingosine via several precursor molecules [61], thereby introducing more delay between the inhibition of the pathway and reduced ceramide levels. Nevertheless, both inhibitors sensitized melanoma cells for CAP treatment, suggesting that pharmacological and CAP-induced reduction in ceramide levels synergize to trigger apoptotic cell death. These findings were then validated ex vivo using the transgenic Tg(Grm1)EPv melanoma mouse model. Although a few in vivo studies on the CAP treatment of tumors exist [62,63], we performed all experiments ex vivo since it allows for a more controlled experimental setup, and no negative consequences were to be expected. This decision was backed by the 3R guiding principles for the ethical use of animals in scientific research [64]. We stained the tumor tissue for cleaved caspase 3 and detected a significant increase after CAP treatment, which was even stronger upon the addition of amitriptyline, thereby validating our in vitro findings. This also shows the first published application of CAP technology in the Tg(Grm1)EPv melanoma mouse model. Previous in vivo studies on CAP-treated melanoma used subcutaneous injection of tumor cells [19,63,65], which has some limitations in reflecting biological tumor development [66,67]. To our knowledge, this study is the first to describe the CAP treatment of transgenic melanoma mouse models, thus facilitating the use of such mice in future research.

## 5. Conclusions

Taken together, we identified a novel mechanism of CAP-induced antitumor activity in melanoma cells involving an activation of the UPR due to protein aggregation and impaired protein folding mechanisms. Endogenous ceramides are drastically reduced at the same time, which could be linked to UPR activation. Based on our findings, we propose that ceramide loss occurs before CAP-induced protein aggregation and calcium release from the endoplasmic reticulum. This is supported by several studies reporting effects of reduced ceramide levels on the structure and aggregation of certain proteins [68,69] and calcium channels [48,70,71]. Furthermore, ceramide loss might increase membrane fluidity, which was previously associated with a higher cellular susceptibility to oxidative stress [72,73]. All findings of this study are illustrated in the graphical abstract. They contribute to a better understanding of CAP and facilitate its potential use in adjuvant tumor therapy. Furthermore, here we demonstrate that the antitumor activity of CAP may be enhanced by applying pharmacological inhibitors of ceramide metabolism.

## Figures and Tables

**Table 1:** Oligonucleotides used as primers for real-time PCR.

Gene	Primer Sequence
18s for	TCTGTGATGCCCTTAGATGTCC
18s rev	CCATCCAATCGGTAGTAGCG
CHOP for	AATGAACGGCTCAAGCAGGA
CHOP rev	AGCCACTTCTGGGAAAGGTG
XBP1s for	CTGAGTCCGCAGCAGGTG
XBP1u for	TGAGAGGTGCTTCCTCGATT
XBP1s/u rev	CACTCAGACTACGTGCACCTCT

## Data Availability

All data are available from the corresponding author upon reasonable request.

## Supplementary Materials

The following supporting information can be downloaded at: https://www.mdpi.com/article/10.3390/cancers15041064/s1, Figure S1: CAP treatment of Mel Im melanoma cells triggers UPR, Figure S2: Increased ceramide levels attenuate CAP effects on Mel Im cells, Figure S3: Pharmacological inhibition of ceramide metabolism in Mel Im cells, File S1: All original Western blot images.
