# Supplementary material for: Cold Atmospheric Plasma Triggers Apoptosis via the Unfolded Protein Response in Melanoma Cells
Source: Cancers (Basel). 2023 Feb 7;15(4):1064. doi: 10.3390/cancers15041064 (PMC9954601; doi:10.3390/cancers15041064)
Supplement: Supplementary file 1 [file cancers-15-01064-s001.zip › cancers-2188979-supplementary.pdf]

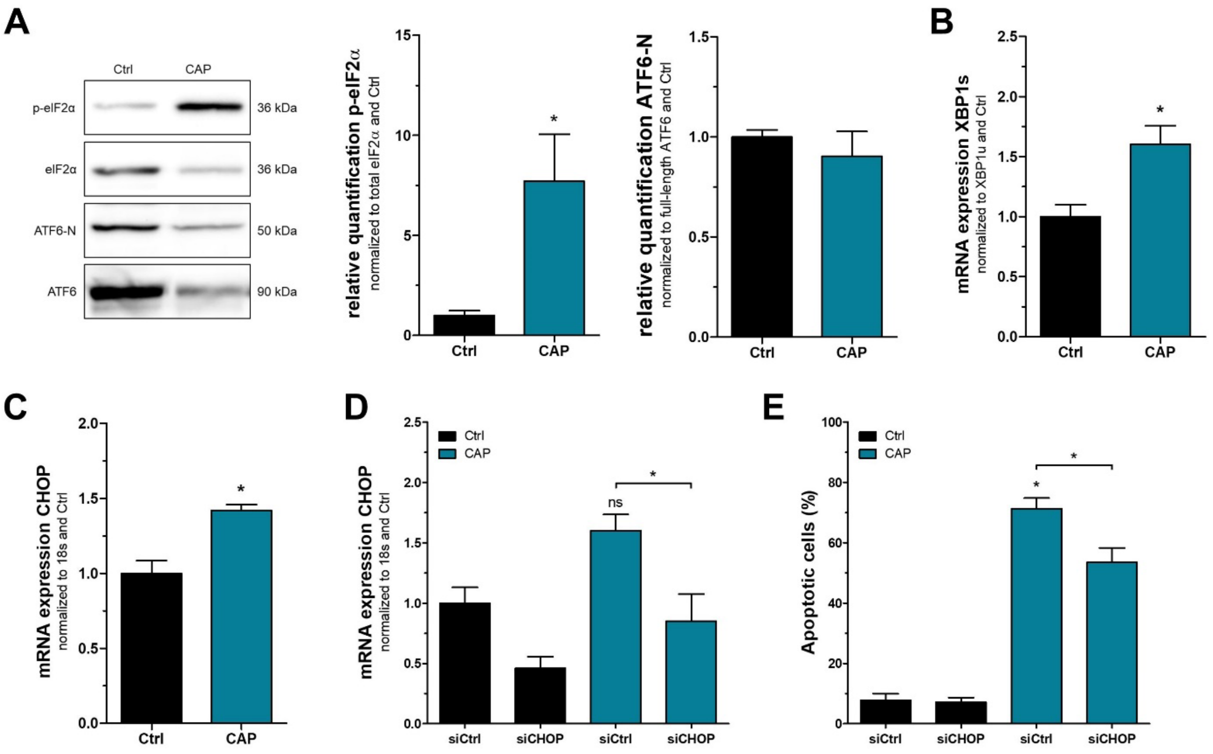

Figure S1: CAP treatment of Mel Im melanoma cells triggers UPR. (**A**) Western blot analysis of phosphorylated eIF2α normalized to total eIF2α protein, and active ATF6-N compared to its inactive variant ATF6. (**B, C**) Expression analysis of spliced XBP1s normalized to unspliced XBP1u, and mRNA expression of pro-apoptotic CHOP. (**D, E**) Knockdown of CHOP mediated by siRNA pools. Expression analysis of CHOP and quantification of apoptotic cells using flow cytometry. Only relevant statistical comparisons are shown in the graphs, including Ctrl siCtrl vs. CAP siCtrl, as indicated by arterisks directly above error bars, and CAP siCtrl vs. CAP siCHOP, as shown by the bracket (n=4 in Figure 1C, all other experiments n=3, *$p < 0.05$). All original Western blot images can be found in the supplementary materials.

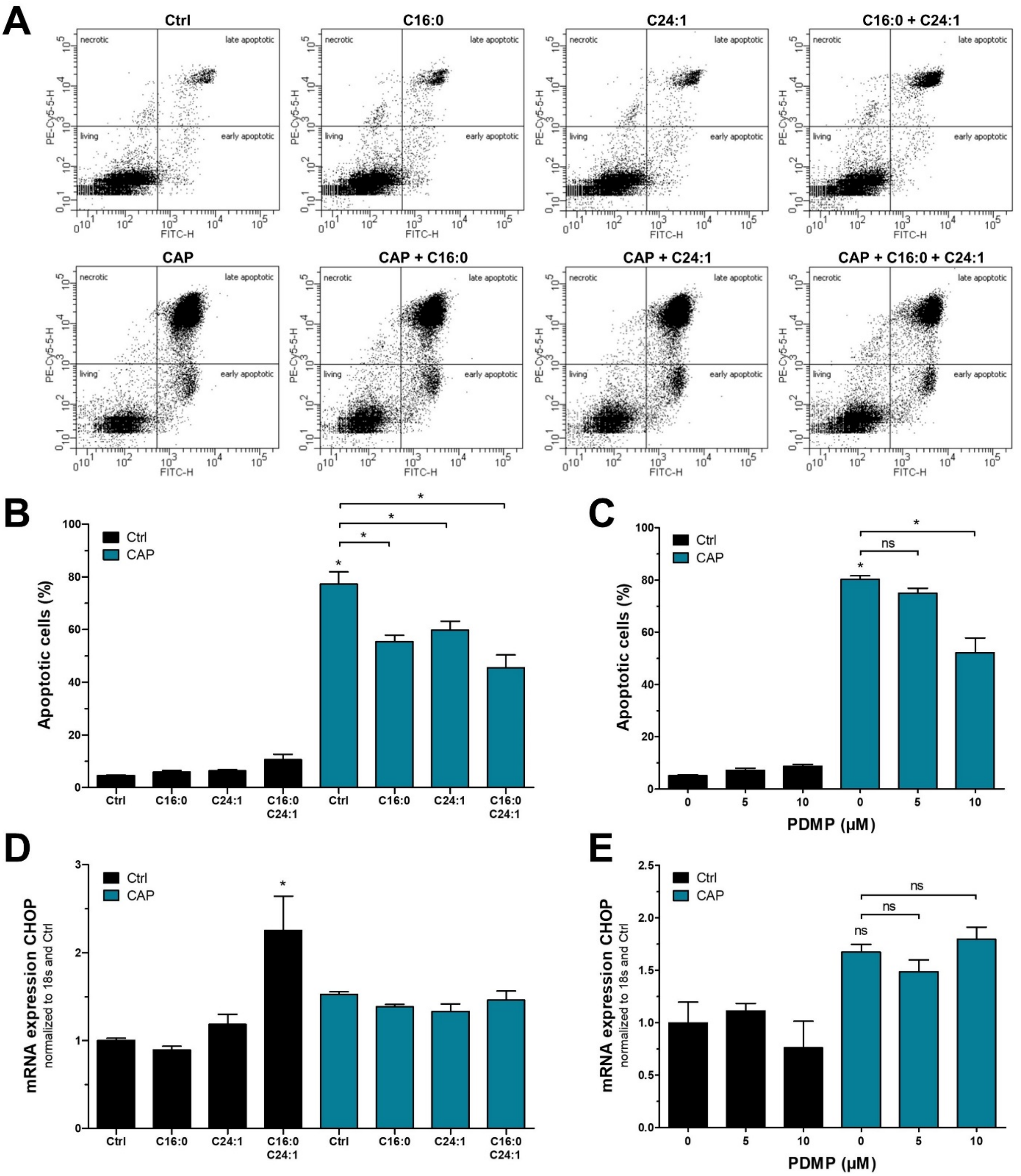

Figure S2: Increased ceramide levels attenuate CAP effects on Mel Im cells. (**A**) Representative images flow cytometric detection of apoptotic cells using Annexin V-FITC and PI staining. (**B, C**) Quantification of apoptotic cells after addition of C16:0 and C24:1 ceramide or PDMP in combination with CAP. (**D**, **E**) Expression analysis of CHOP after combined treatment with CAP and either ceramides or PDMP (n = 3, *p < 0.05, ns = not significant).

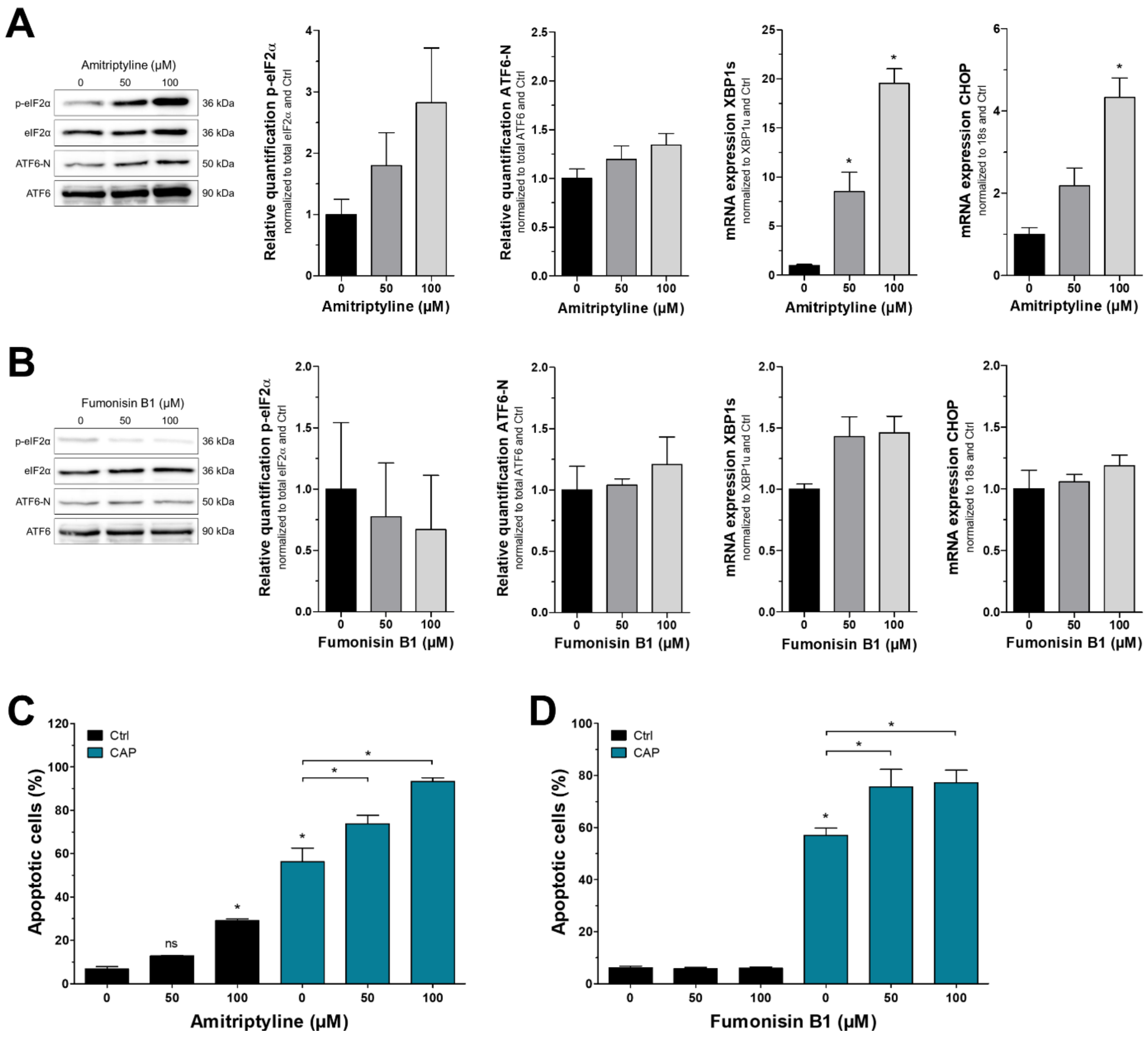

Figure S3: Pharmacological inhibition of ceramide metabolism in Mel Im cells. Analysis of UPR activation after 1 h treatment with (**A**) amitriptyline, an inhibitor of acid sphingomyelinase, or (**B**) fumonisin B1 which inhibits ceramide synthases, in Mel Im cells. UPR activation was assessed using several protein and mRNA markers. (**C**, **D**) Flow cytometric quantification of apoptosis after the same treatment in combination with CAP ($n = 3$, $^*p < 0.05$). All original Western blot images can be found in the supplementary materials.
