# Supplementary material for: Cold Atmospheric Plasma Triggers Apoptosis via the Unfolded Protein Response in Melanoma Cells
Source: Cancers (Basel). 2023 Feb 7;15(4):1064. doi: 10.3390/cancers15041064 (PMC9954601; doi:10.3390/cancers15041064)
Supplement: Supplementary file 1 [file cancers-15-01064-s001.zip › Supplementary File S1.pdf]

Blot 1: **Figure 1A**

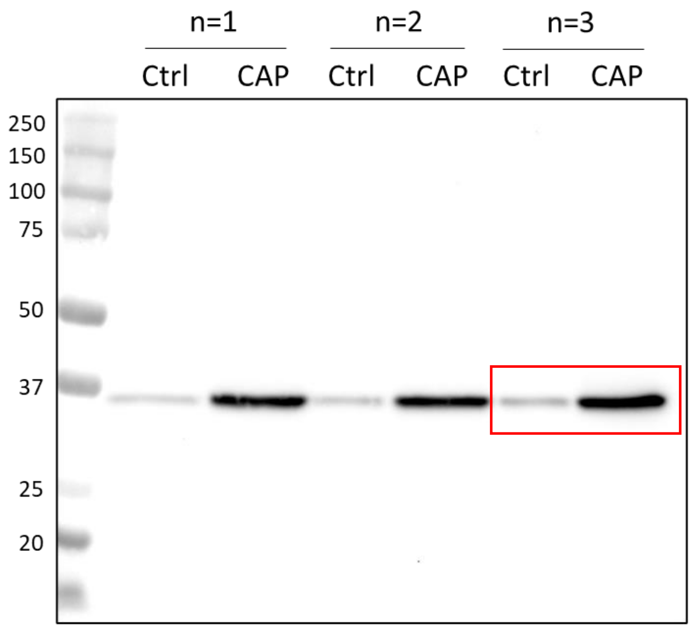

p-eIF2alpha (36 kDa)

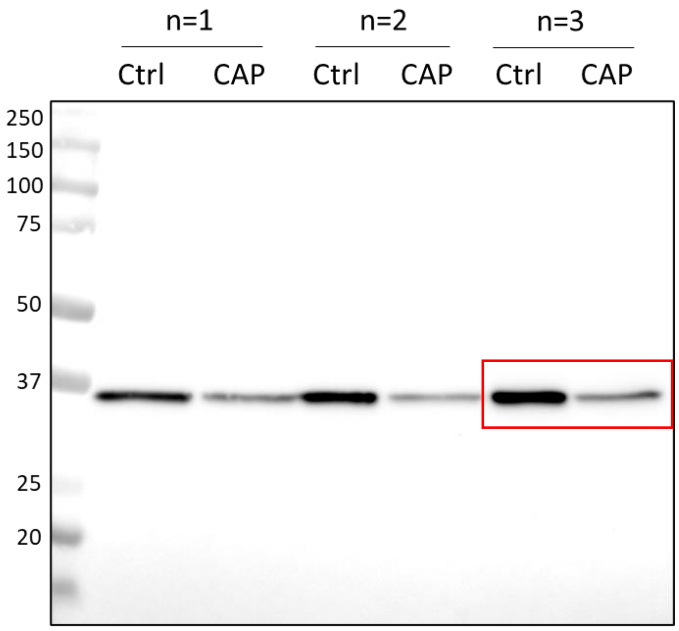

eIF2alpha (36 kDa)

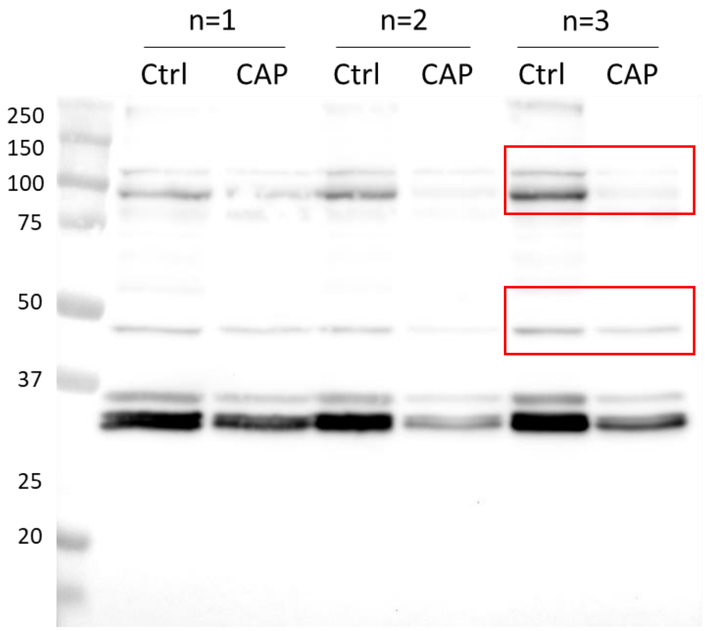

ATF6 (active: 50 kDa, inactive: 90 kDa)

Blot 2: **Figure 5A** n=1

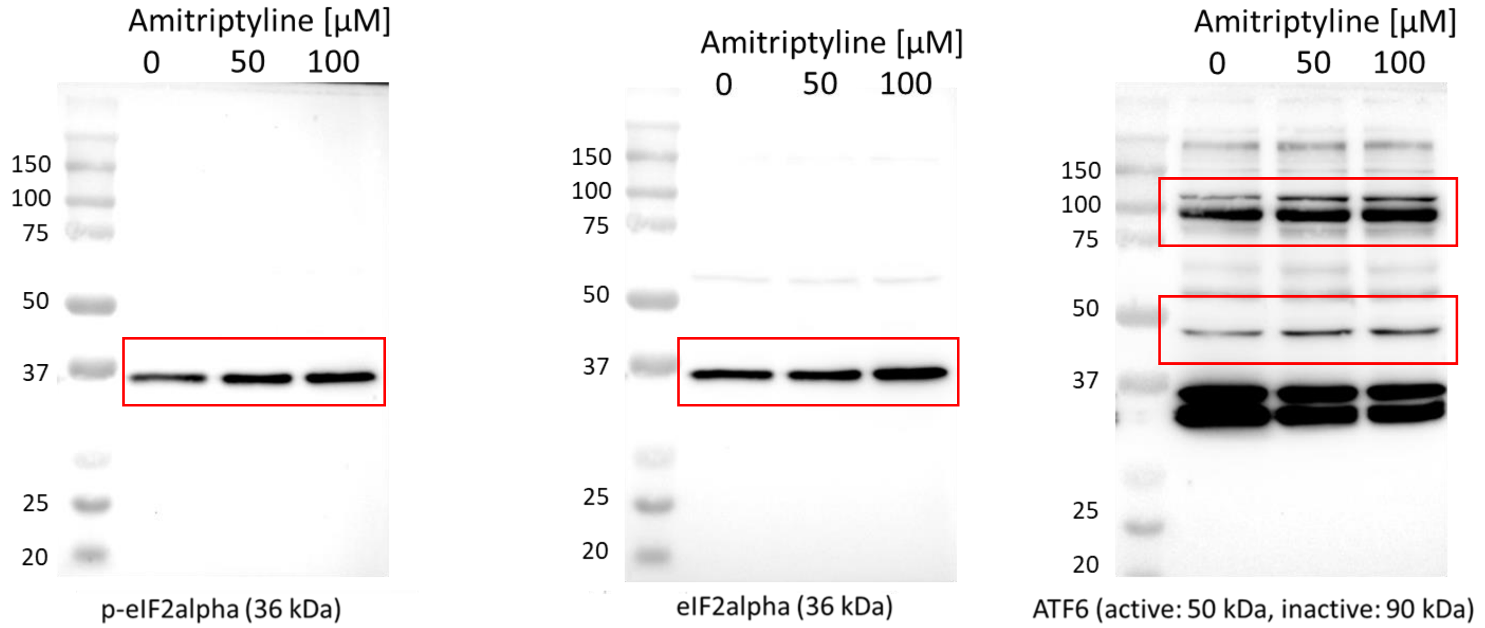

Blot 3: **Figure 5A** n=2 (Samples 1 µM, 5 µM and 10 µM were not included in the analysis)

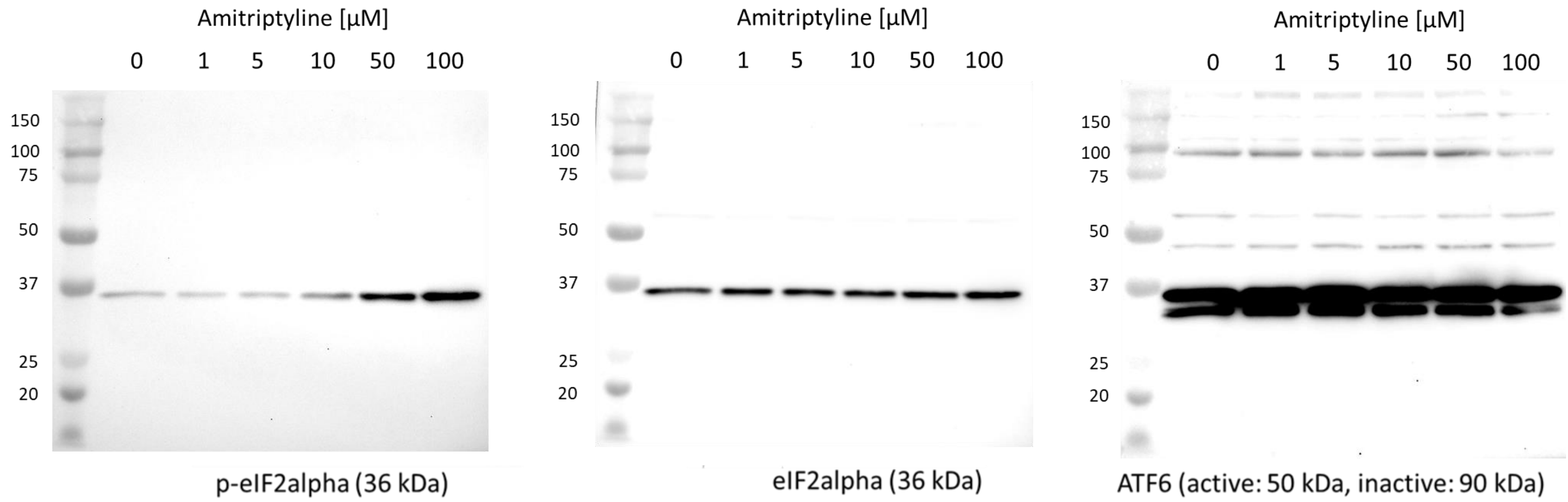

Blot 4: **Figure 5A** n=3 (Samples 1 µM, 5 µM and 10 µM were not included in the analysis)

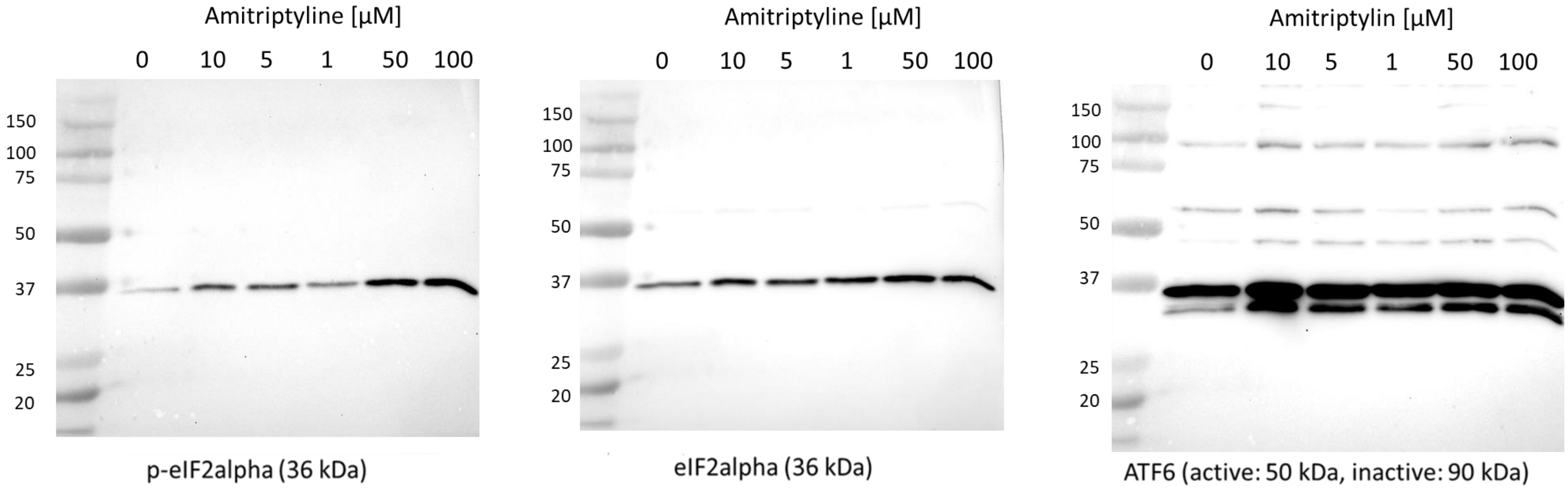

Blot 5: **Figure 5B** n=1 (Samples 1 µM, 5 µM and 10 µM were not included in the analysis)

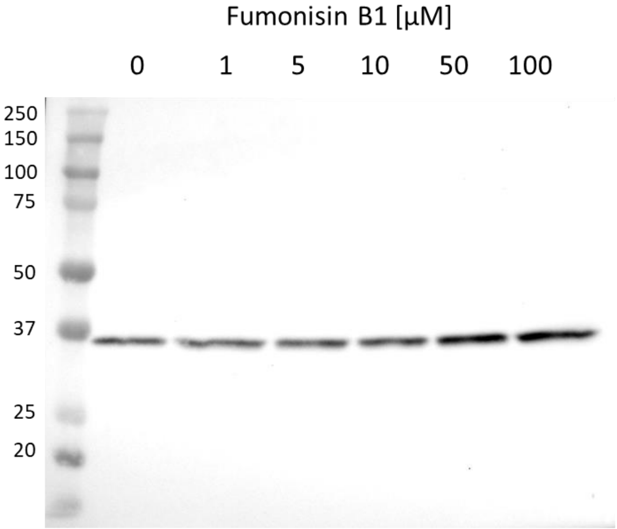

p-eIF2alpha (36 kDa)

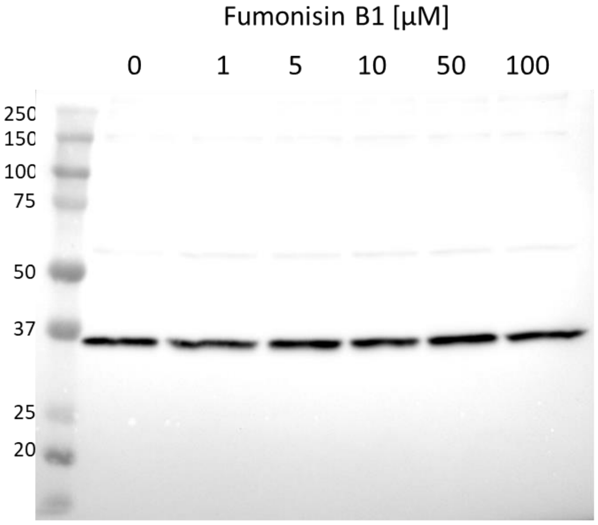

eIF2alpha (36 kDa)

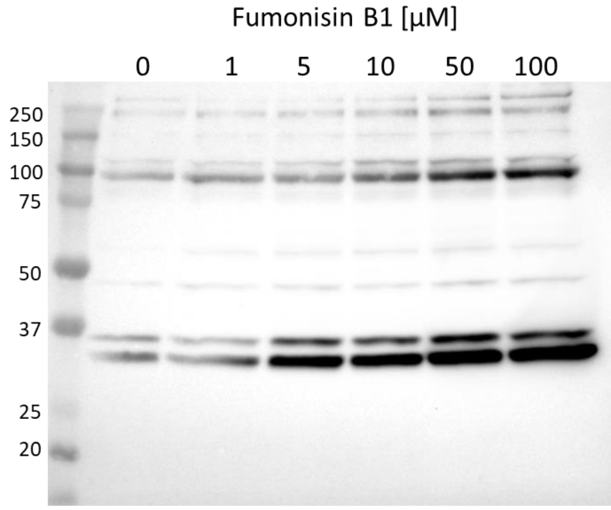

ATF6 (active: 50 kDa, inactive: 90 kDa)

Blot 6: **Figure 5B** n=2

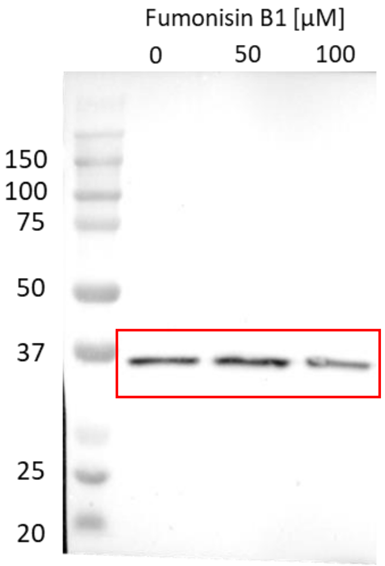

p-eIF2alpha (36 kDa)

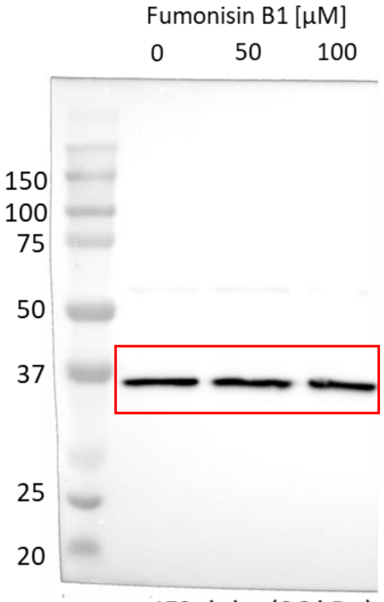

eIF2alpha (36 kDa)

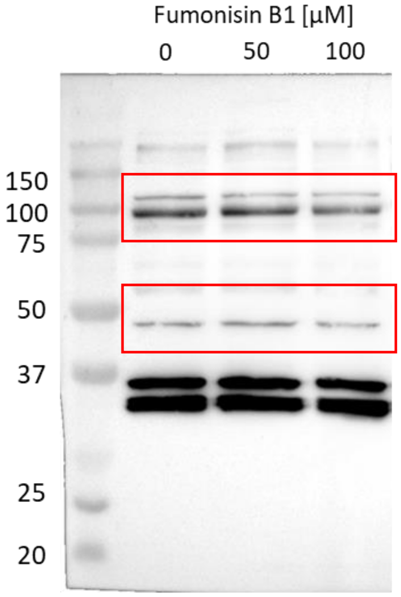

ATF6 (active: 50 kDa, inactive: 90 kDa)

Blot 7: **Figure 5B** n=3 (Samples 1 µM, 5 µM and 10 µM were not included in the analysis)

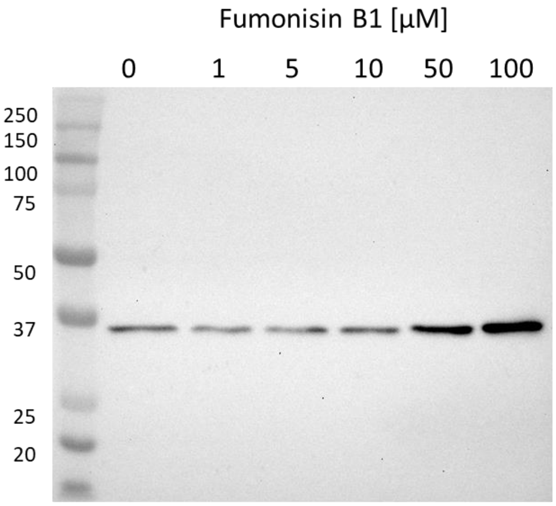

p-eIF2alpha (36 kDa)

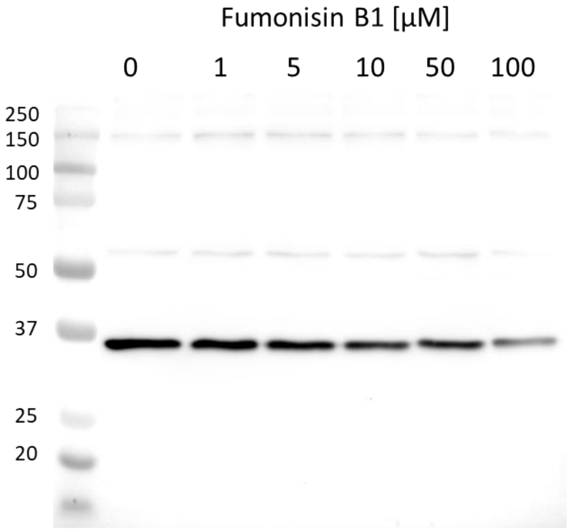

eIF2alpha (36 kDa)

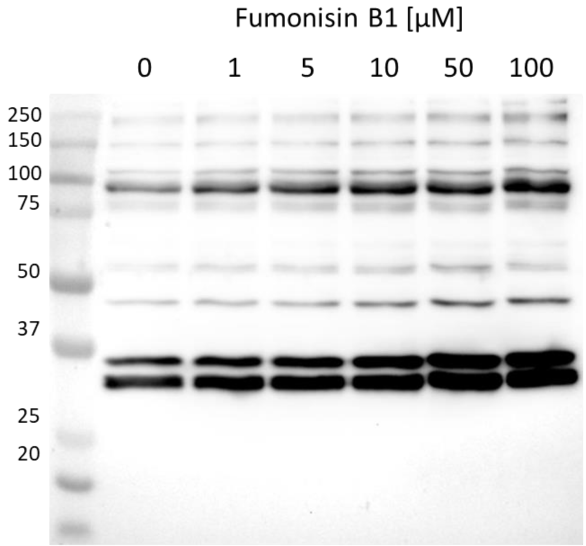

ATF6 (active: 50 kDa, inactive: 90 kDa)

Blot 8: **Figure S1A**

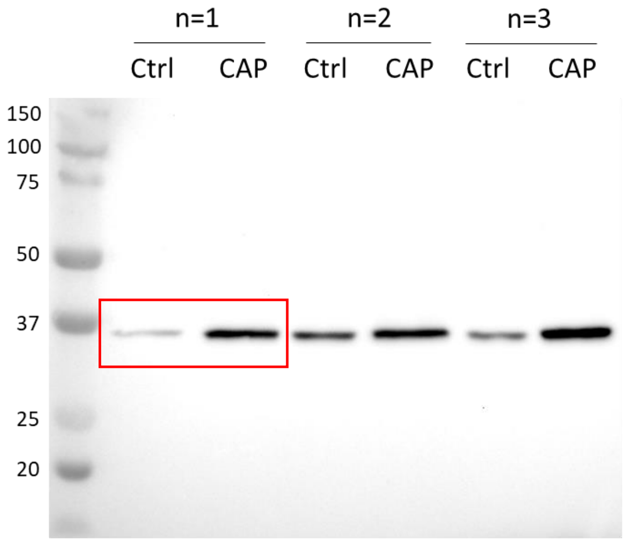

p-eIF2alpha (36 kDa)

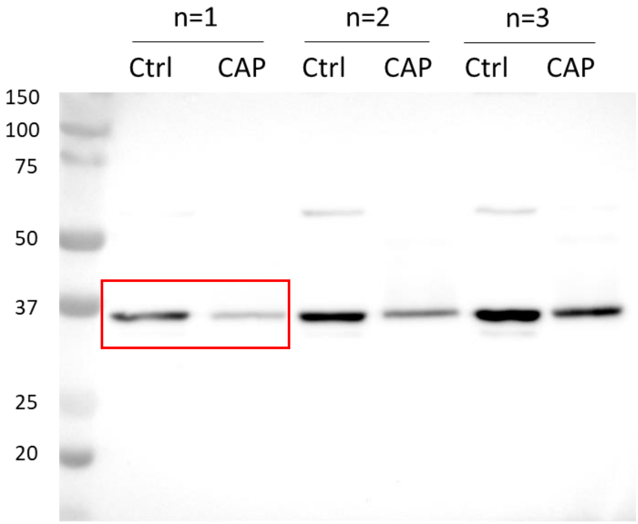

eIF2alpha (36 kDa)

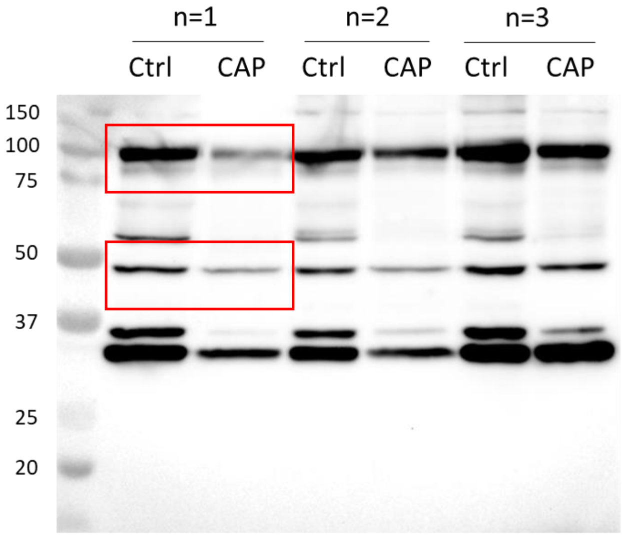

ATF6 (active: 50 kDa, inactive: 90 kDa)

Blot 9: **Figure S3A n=1** (Samples 1 µM, 5 µM and 10 µM were not included in the analysis)

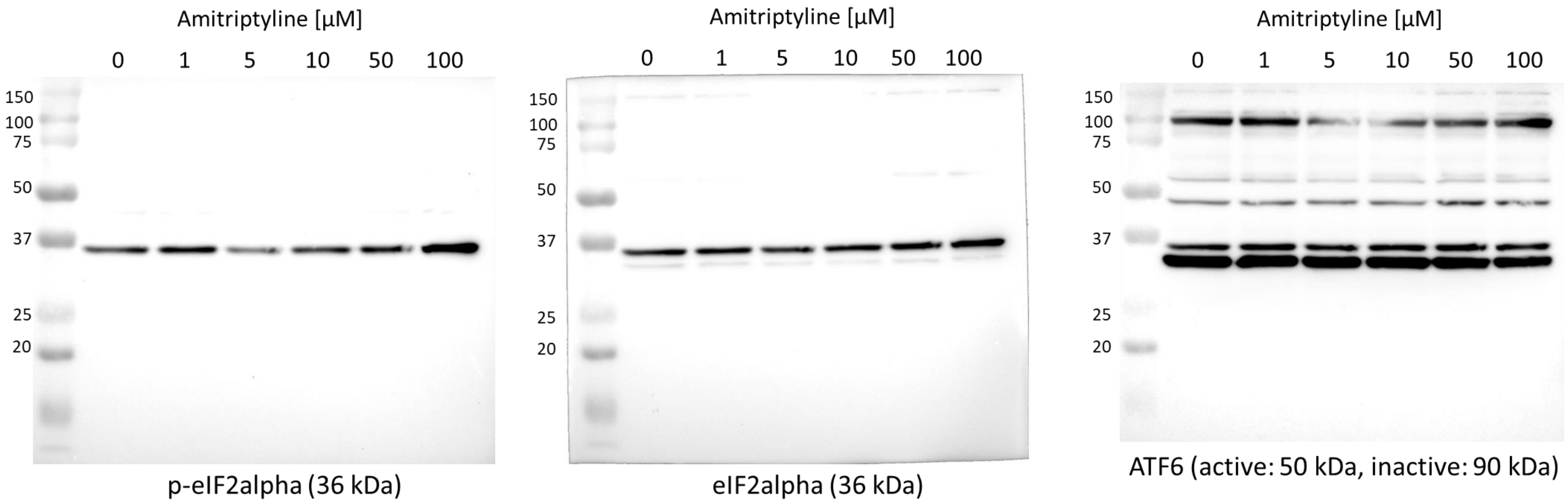

p-eIF2alpha (36 kDa) eIF2alpha (36 kDa) ATF6 (active: 50 kDa, inactive: 90 kDa)

Blot 10: **Figure S3A n=2** (Samples 1 µM, 5 µM and 10 µM were not included in the analysis)

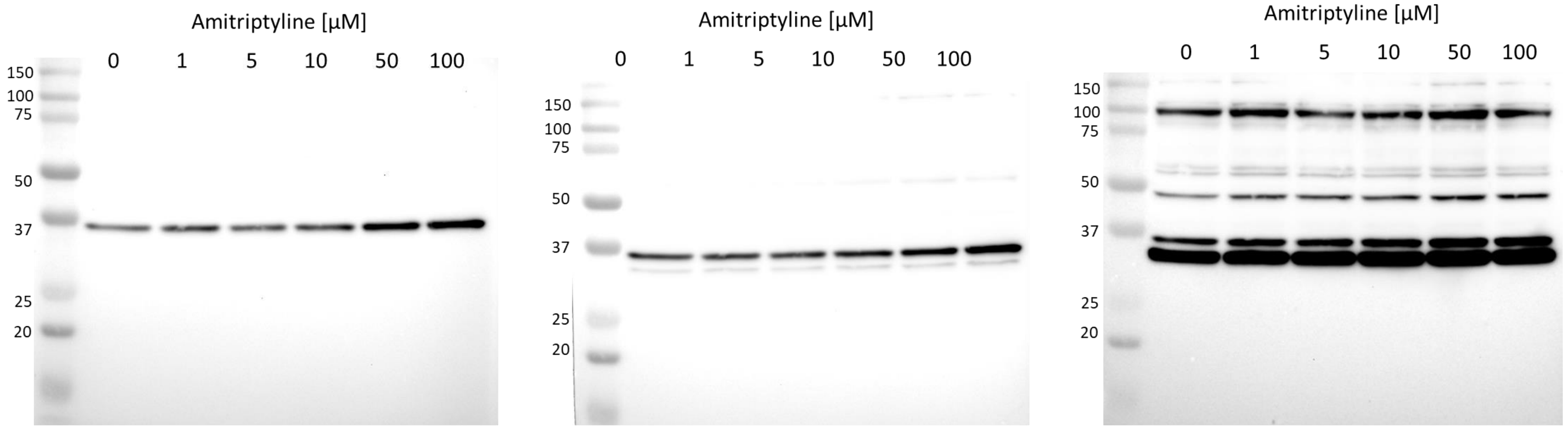

p-eIF2alpha (36 kDa) eIF2alpha (36 kDa) ATF6 (active: 50 kDa, inactive: 90 kDa)

Blot 11: **Figure S3A n=3**

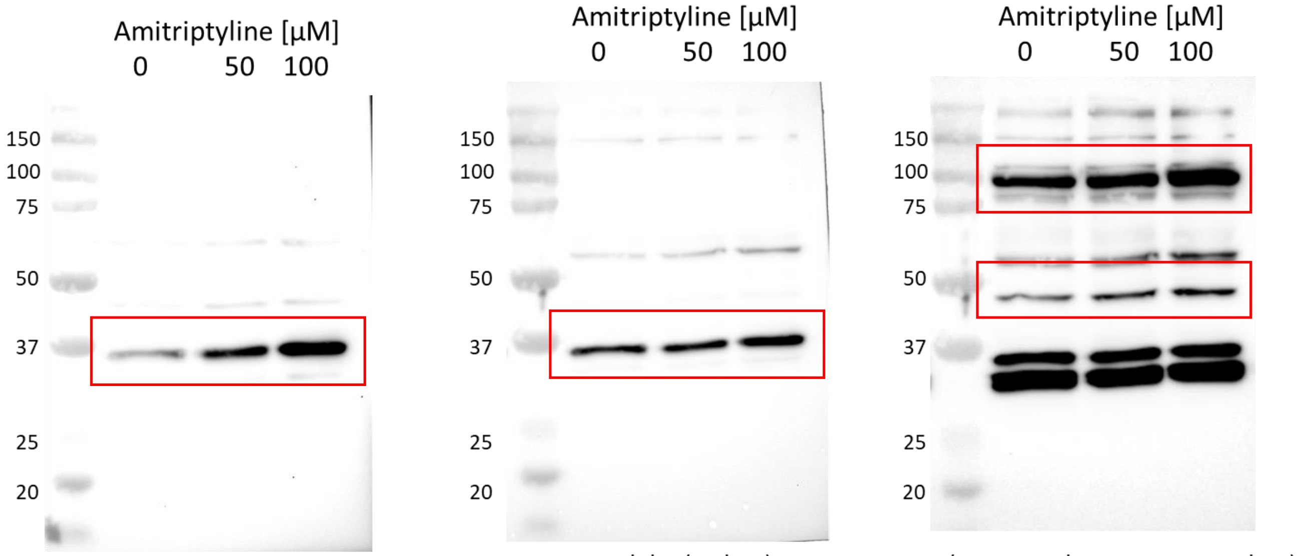

p-eIF2alpha (36 kDa) eIF2alpha (36 kDa) ATF6 (active: 50 kDa, inactive: 90 kDa)

Blot 12: **Figure S3B n=1** (Samples 1 µM, 5 µM and 10 µM were not included in the analysis)

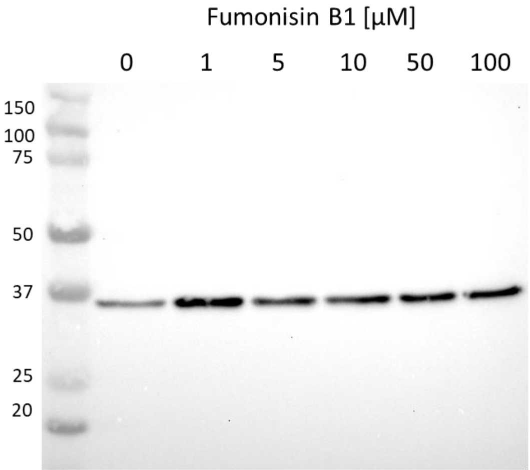

p-eIF2alpha (36 kDa)

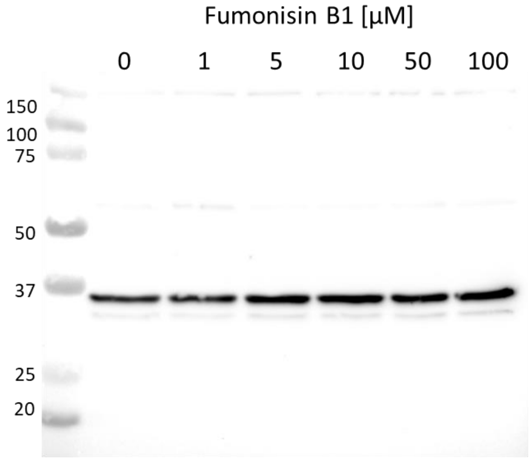

eIF2alpha (36 kDa)

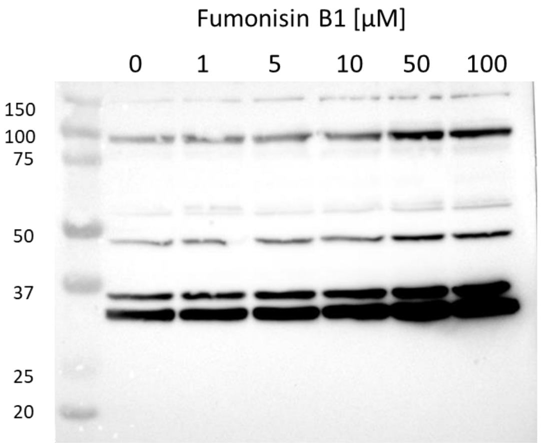

ATF6 (active: 50 kDa, inactive: 90 kDa)

Blot 13: **Figure S3B n=2** (Samples 1 µM, 5 µM and 10 µM were not included in the analysis)

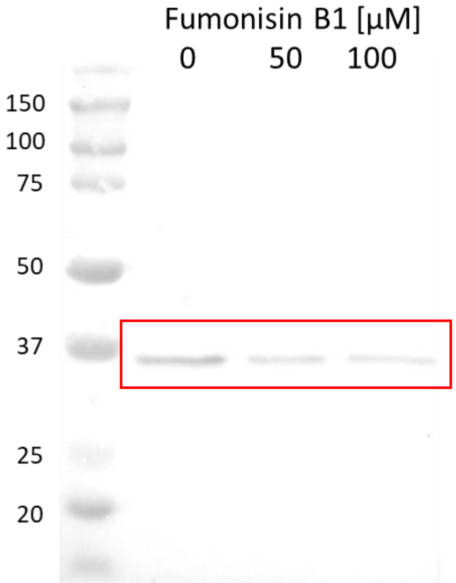

p-eIF2alpha (36 kDa)

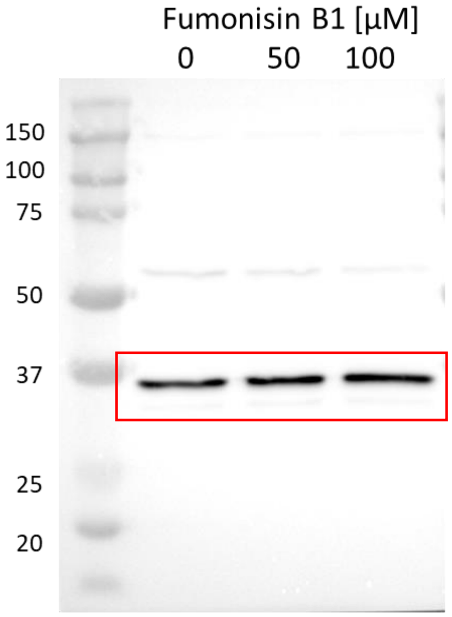

eIF2alpha (36 kDa)

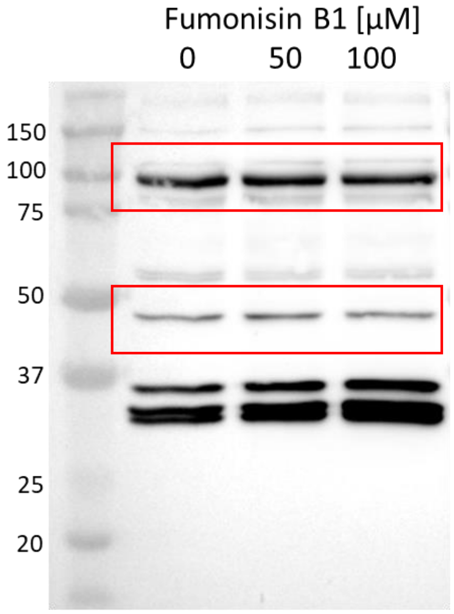

ATF6 (active: 50 kDa, inactive: 90 kDa)

Blot 14: **Figure S3B n=3** (Samples 1 µM, 5 µM and 10 µM were not included in the analysis)

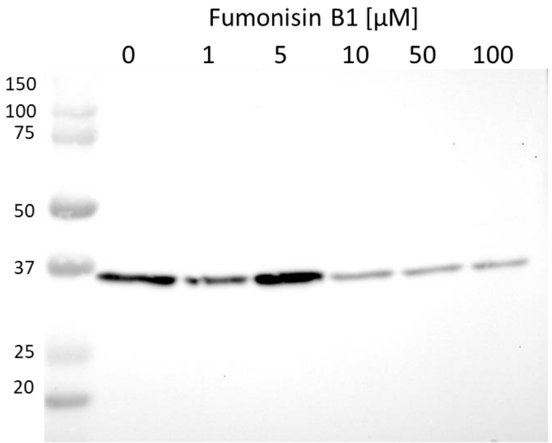

p-eIF2alpha (36 kDa)

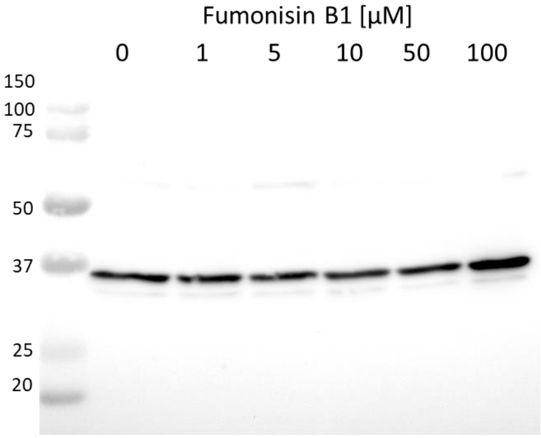

eIF2alpha (36 kDa)

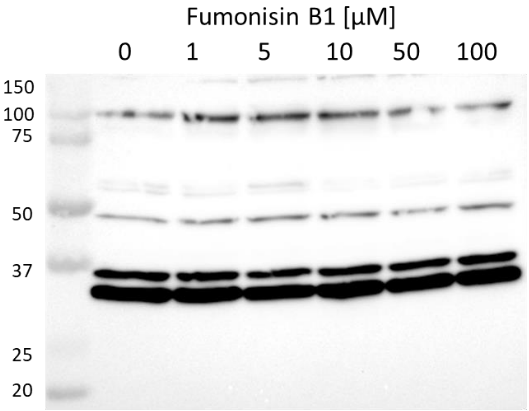

ATF6 (active: 50 kDa, inactive: 90 kDa)
